# Development of betabodies: The next generation of phosphatidylserine targeting agents

**DOI:** 10.1016/j.jbc.2024.107681

**Published:** 2024-08-17

**Authors:** Natalie Z. Phinney, Xianming Huang, Jason E. Toombs, Rolf A. Brekken

**Affiliations:** 1 Department of Surgery, UT Southwestern Medical Center, Dallas, Texas, USA; 2 Hamon Center for Therapeutic Oncology Research, UT Southwestern Medical Center, Dallas, Texas, USA; 3 Cancer Biology Graduate Program, UT Southwestern Medical Center, Dallas, Texas, USA; 4 Department of Pharmacology, UT Southwestern Medical Center, Dallas, Texas, USA; 5 Harold C. Simmons Comprehensive Cancer Center, UT Southwestern Medical Center, Dallas, Texas, USA

**Keywords:** phosphatidylserine, PS-targeting, fusion protein, β2-glycoprotein 1, tumor microenvironment

## Abstract

Externalized phosphatidylserine (PS) is a phospholipid and a selective marker of the tumor microenvironment (TME). It is exposed on the outer leaflet of the plasma membrane of tumor-associated endothelial cells, apoptotic tumor cells, and some viable tumor cells, where it functions in part to suppress immune responses by binding to PS receptors expressed on tumor-infiltrating myeloid cells. PS has been targeted with antibodies, such as bavituximab, that bind the phospholipid *via* a cofactor, β2-glycoprotein 1 (β2GP1); these antibodies showed excellent specificity for tumor vasculature and induce an immune stimulatory environment. We have advanced this concept by developing the next generation of PS targeting agent, a fusion protein (betabody) constructed by linking PS-binding domain V of β2GP1 to the Fc of an IgG2a. Betabodies bind to externalized PS with high affinity (∼1 nM), without the requirement of a co-factor and localize robustly to the TME. We demonstrate that betabodies are a direct PS-targeting agent that has the potential to be used as anti-tumor therapy, drug delivery vehicles, and tools for imaging the TME.

The major advancement in the last decade in cancer therapy has been the discovery and approval of antibodies that interrupt immune checkpoints (1, 2). Antibodies targeting cytotoxic T-lymphocyte antigen-4 (CTLA-4) and programmed death-1 (PD-1)/programmed death-1 ligand (PDL-1) have improved outcomes for many cancer patients (3, 4, 5, 6, 7, 8, 9). Often, though, cancer patients exhibit low responses to these therapies or rapidly develop resistance (10, 11). The need for additional immunoregulatory targets in the tumor microenvironment (TME) continues to drive the development of new immune-modulatory modalities.

Phosphatidylserine (PS) has been studied in pathological contexts and has emerged as a viable candidate for enhancing anti-tumor immune responses (12). PS is a negatively charged phospholipid that is part of the structure of the lipid bilayer in all cell membranes. It is sequestered to the inner leaflet of the plasma membrane in normal cells unless they become apoptotic, at which point PS is translocated to the outer leaflet to engage with PS receptors on phagocytic cells (13, 14). PS engagement with PS receptors initiates efferocytosis, the clearance of apoptotic cells, and subsequent local immune suppression (15, 16). Cells in the TME have been shown to exploit externalized PS signaling to inhibit the innate and adaptive immune response; environmentally stressed tumor cells, apoptotic and necrotic cells, as well as healthy, activated endothelial cells externalize PS in the TME (17, 18). Macrophages, in particular, express PS receptors and, after binding PS, polarize into an anti-inflammatory phenotype and secrete cytokines that prevent dendritic cell maturation, antigen presentation, and subsequent T-cell priming and activation (12, 19, 20, 21).

PS-binding antibodies have been developed to target externalized PS in the TME (22). This suite of antibodies localizes to the TME and, in particular, tumor vasculature. Multiple studies have demonstrated that PS-targeting antibodies enhance the efficacy of standard therapy and immune therapy for cancer (20, 23, 24, 25, 26, 27). Bavituximab, a chimeric PS-targeting antibody is currently in clinical testing in multiple indications [NCT03519997, NCT04150900, NCT03139916]. However, these PS-targeting antibodies require the serum protein β2-Glycoprotein-1 (β2GP1) and must dimerize two units of β2GP1 to mediate binding to PS (24). Additionally, this dependency impacts the size of the therapeutic structure and its ability to permeate the tumor beyond the vasculature.

Here we report developing the next generation of PS targeting agent, a recombinant fusion protein composed of the PS-binding domain of β2GP1 fused to an IgG2a Fc. This unique fusion protein, significantly smaller in size compared to PS-binding antibodies, binds PS directly with high affinity and specifically localizes to the TME.

## Results

### **β**2GP1 null C57Bl/6 mice demonstrate the function of β2GP1 in targeting PS

**Figure 1:**
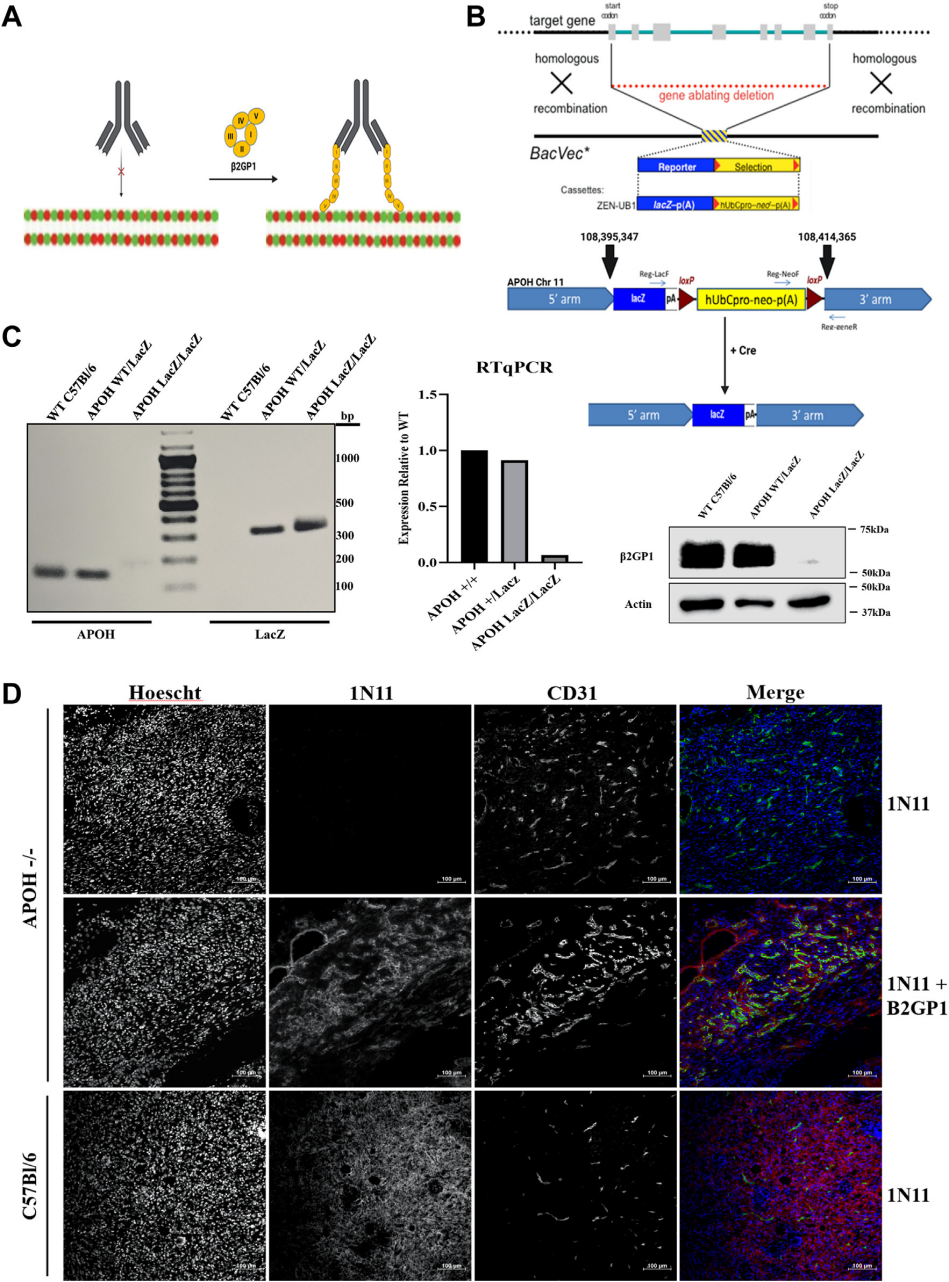
**β2-Glycoprotein 1 is an essential cofactor for binding of PS-targeting antibodies to externalized PS**. *A*, *schematic of PS-targeting antibody binding to PS via β2GP1.* PS-targeting antibodies (*e.g.*, 1N11, 2aG4) require β2-Glycoprotein-1 (β2GP1) to bind to externalized phosphatidylserine (PS) (24). PS-targeting antibodies bind β2GP1 resulting in dimerizing of β2GP1 which binds to PS *via* domain V. Dimerization of β2GP1 increases its affinity for PS from 1 μM to 1 nM. *B*, *schematic for deletion of the Apoh gene*. *C*, Apoh^−/−^ mice were verified by RT-PCR and Western blotting. Litters from an N7 Apoh^+/−^ x N7 Apoh^+/−^ cross were genotyped using primer sequences provided by the KOMP repository for the Apoh WT gene and the lacZ cassette, indicating gene loss. Pups positive for lacZ and negative for Apoh WT were retained as Apoh^−/−^ and bred. The genotyping was further verified by RT-PCR and Western blot of liver lysates from a C57Bl/6 WT mouse, an Apoh^+/−^ heterozygous mouse, and an Apoh^−/−^ mouse. *D*, *in vivo localization of PS targeting antibodies*. Human PS-targeting antibody, 1N11 (50 μg), was injected i.v. into KPfC tumor bearing mice, with three groups: 1N11 into Apoh^−/−^ mice, 1N11 preincubated with β2GP1into Apoh^−/−^ mice, and 1N11 into C57Bl/6 WT mice (n = 3). After 2 h of circulation, tumors were resected and snap frozen for IF staining of 1N11 localization.

A drawback of the PS-targeting antibodies is that they do not bind PS directly. PS binding requires two units of a cofactor, β2GP1 (23) (Fig. 1*A*). We developed β2GP1 (*Apoh*) deficient mice on a C57Bl/6 background to demonstrate the essential relationship between PS-targeting antibodies and β2GP1 *in vivo*. Mice were created as described in the *Experimental Procedures* resulting in the complete deletion of the APOH gene *via* insertion of the LacZ cassette (Fig. 1*B*). Homozygous Apoh knockout mice were verified by genotyping for the WT gene and LacZ cassette; they were further characterized *via* qPCR of RNA extracted from mouse livers and Western blot for β2GP1 protein in liver lysates (Fig. 1*C*).

### Dimerized **β**2GP1 is an essential cofactor for PS-targeting antibodies to bind to externalized PS and localization to the tumor microenvironment

To demonstrate the requirement of β2GP1 for binding to PS, we utilized *Apoh*^−/−^ mice. *Apoh*^−/−^ mice and WT C57Bl/6 mice bearing established subcutaneous mouse pancreatic tumors (KPfC) were injected i.v. with 1N11 alone or 1N11 preincubated with purified β2GP1. After 2 h, mice were euthanized and perfused, and tumors were resected and snap-frozen. Frozen tumor sections were stained for 1N11 localization to the TME; sections were also stained for CD31, a marker of endothelial cells to show localization of the antibody to tumor vasculature as demonstrated prior (28). Immunofluorescence (IF) staining of each group illustrated that 1N11 localizes to tumor vasculature in a β2GP1-dependent manner, showing no localization in tumor-bearing *Apoh*^−/−^ mice without prior incubation of 1N11 with a 2 to 1 M ratio of β2GP1 (Fig. 1*D*).

### Evolution of betabodies from PS-targeting antibodies to a single domain Fc fusion protein

To develop direct PS binders, full-length β2GP1 was initially fused to an IgG Fc domain (FLB). A concern with this design was the size (150.6 kDa) and potential toxicity. Anti-phospholipid antibodies, the causative agent in antiphospholipid syndrome (APS) typically bind to domain I of β2GP1 (29, 30, 31, 32, 33, 34, 35). Although a small number of studies have claimed that there are some endogenous anti-β2GP1 antibodies that target domains II-IV, pathological antibodies to β2GP1 isolated from human APS patients only interact with domain I (31). PS-targeting antibody 1N11 is able to diminish APS pathology by interfering with APS antibody-β2GP1 interaction (36).

**Figure 2:**
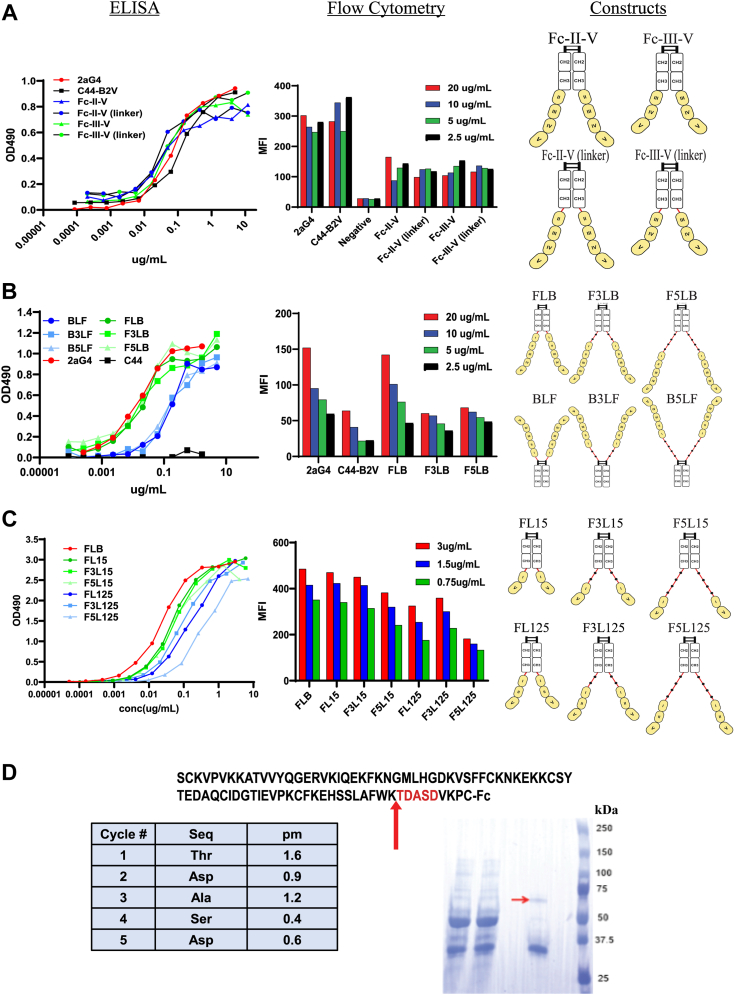
**Betabodies evolved from full-length β2GP1 fusion proteins to small single-domain constructs**. *A–C*, betabodies containing only partial β2GP1 sequences, Fc-II-V and Fc-III-V (*A*), full-length β2GP1 fused to either N or C terminus of the Fc (*B*), domains I and V or I, II, and V contain 1, 3, or 5 linkers (*C*) and C44-B2V (IgG2a isotype control antibody linked to domain V) and 2aG4 Ab were tested for binding to PS by ELISA and flow cytometry on irradiated NS0 cells (*A*) or Daudi cells (*B* and *C*). Constructs were evaluated for PS binding on cells by comparing mean fluorescence intensity (MFI). *D*, identification of the betabody cleavage site by N-terminal sequencing was performed on Domain I/V betabodies recovered from *in vivo* circulation in SCID mice.

We tested multiple variations of β2GP1 domains fused to Fc (Table 1) *in vitro* using a PS-binding ELISA assay and flow cytometry to show binding to cells. For flow cytometry, cells were irradiated or treated with H_2_O_2_, as indicated, to induce PS externalization. To determine which domains of β2GP1 were optimal for binding to PS, we produced constructs lacking domain I. These “domain betabodies” contained domains II-V or III-V fused to Fc. Two constructs, Fc-II-V and Fc-III-V, with and without a linker were transiently expressed in CHO cells and tested. Each construct bound PS with activity similar to 2aG4, a mouse PS-targeting antibody (18) as shown by ELISA (Fig. 2*A*, left panel); however, they displayed significantly worse activity than 2aG4 on irradiated NS0 cells quantified by flow cytometry (Fig. 2*A*, right panel). Next, we tested constructs consisting of the full-length wild-type mouse β2GP1 fused to the N-terminus (BLF) or the C-terminus (FLB) of IgG2a Fc, as well as versions with multiple linker lengths: GGGGS – BLF, FLB; (GGGGS)_3_- B3LF, F3LB; (GGGGS)_5_- B5LF, F3LB (Table 1). The C-terminal constructs bound to PS *via* ELISA similarly to 2aG4 and with higher affinity than the N-terminal versions (Fig. 2*B*, left panel). Flow cytometry analysis of irradiated cells revealed that only the C-terminal construct containing a single linker (FLB) binds to PS exposed on the cell surface similarly to 2aG4 (Fig. 2*B*, right panel). Subsequent studies investigated the pharmacokinetics of the FLB constructs *in vivo*. These constructs were cleared in approximately 30 min. Hypothesizing that the heavy glycosylation pattern of domains II and III of β2GP1 may be causing clearance in the liver (37), https://www.uniprot.org/uniprotkb/Q01339/entry#function, we performed a 10-min PK study and found that the majority of FLB had already accumulated in the liver (Fig. S1*A*). Subsequent de-glycosylation of domains II and III of β2GP1 chemically with PNGase F or genetically (dgFLB, dgF3LB, dgF5LB) resulted in significantly diminished PS binding (Fig. S1*B*). In summary, we found that the C-terminal Fc-β2GP1 constructs bound PS-positive cells as efficiently as 2aG4 but were cleared quickly by the liver *in vivo*.

**Table 1:** Evolving betabody constructs

Betabody Construct	β2-Glycoprotein-1 domains	N- or C-terminal fusion	Linkers	Mutations
FLB	1–5	C	1	-
F3LB	1–5	C	3	-
F5LB	1–5	C	5	-
BLF	1–5	N	1	-
B3LF	1–5	N	3	-
B5LF	1–5	N	5	-
Fc-II-V	2–5	C	1	-
Fc-III-V	3–5	C	1	-
dgFLB	1–5	C	1	N86Q; N98Q; N143Q; N164Q; N174Q
dgF3LB	1–5	C	3	N86Q; N98Q; N143Q; N164Q; N174Q
dgF5LB	1–5	C	5	N86Q; N98Q; N143Q; N164Q; N174Q
FL15^a^	1,5	C	1	-
F3L15	1,5	C	3	-
F5L15	1,5	C	5	-
FL125	1,2,5	C	1	N86Q; N98Q
F3L125	1,2,5	C	3	N86Q; N98Q
F5L125	1,2,5	C	5	N86Q; N98Q
KL15^a^	1,5	C	1	-
SL15	1,5	C	1	K308S
KL5	5	C	1	-

a Constructs are the same.

As a result of the abrupt clearance of the FLB constructs *in vivo* due to the glycosylation of domains II and III, we returned to the truncated domain betabodies. We produced betabodies that fused domains I and V (FL15, F3L15, F5L15) or domains I, II, and V (FL125, F3L125, F5L125) with 1, 3, or 5 linkers, respectively (Table 1). The glycosylation sites in domain II were mutated (N86Q and N98Q) to eliminate the clearance issue. All six constructs were tested for PS binding *in vitro*; FL15, F3L15, and F5L15 bound PS by ELISA only marginally less effectively than FLB, whereas the I, II, V constructs bound to PS significantly less in comparison (Fig. 2*C*, left panel). Flow cytometry analysis showed that I, V constructs bound externalized PS at the same rate as FLB, but, again, the I, II, and V constructs showed much less effective binding (Fig. 2*C*, right panel). Interestingly, the number of linkers did not make a significant difference functionally, though as more linkers were included, there was a slight reduction in binding. In conclusion, FL15, a C-terminal fusion containing β2GP1 domains I and V and a single linker of the Fc and β2GP1 domains proved to be just as effective in binding PS by ELISA and flow cytometry as the Fc-β2GP1 construct.

### Point mutations in domain V are required to maintain stability *in vivo*

Because one of the residues (K308) in β2GP1 domain V is involved in binding to LDL receptor ApoER2 and these interactions have been implicated in the pathogenicity of APS (38), we introduced a point mutation K308A, K308D, and K308S and generated three variants of FL15. From this point, the non-mutated version will be referred to as KL15, where K denotes K308. FL15 mutants K308A, K308D, and K308S will be referred to as AL15, DL15, or SL15, respectively; all variants, including KL15, bound PS in the ELISA equally well, except for AL15 (K308A) which completely lost binding activity (Fig. S1*C*). By flow cytometry, KL15 bound irradiated cells better than the other variants, though SL15 (K308S) had the closest binding pattern to KL15 (Fig. S1*D*). We then proceeded to test the pharmacokinetics of KL15 and SL15 *in vivo* by injecting ^125^I-labled KL15 or SL15 *via* the tail vein. Blood was sampled at multiple time points and ^125^I counts were determined with a γ-counter. KL15, at approximately 125 h, had a longer half-life than SL15 (Fig. S2*A*), and both lasted longer *in vivo* than bavituximab (∼48 h) (39), a chimeric PS-targeting antibody that is currently in clinical testing. KL15 was the most promising construct *in vitro* and *in vivo* to this point. Given the potential APS-like toxicity due to domain I, we also produced and characterized the smaller KL5, a construct with the same structure as KL15 but only including domain V (Table 1).

Subsequent PK studies with KL5 and KL15 identified that each betabody was losing PS binding efficacy *in vivo*. For example, after overnight circulation in SCID mice, the PS binding of KL15 was reduced to 65%. KL5 fared better with a PS-binding efficacy of ∼60 to 70% after 3 days in SCID mice (Fig. S2*B*). Next, purified KL15c was added to either freshly collected plasma or serum from naïve mice, incubated for indicated time periods, and analyzed for PS-binding activity by PS ELISA. Results indicate that KL15c is stable in plasma but relatively unstable in serum over time (Fig. S2*C*). KL5c and KL15c fragmentation/degradation was confirmed by SDS-PAGE 24 h after injecting these betabodies into SCID mice (Fig. S2*D*). To identify the sites of possible degradation affecting PS-binding, we repeated *in vivo* PK with an n-terminal betabody (KL15n); it was purified from the plasma by protein A, run on SDS-PAGE, and submitted for N-terminal sequencing to identify the site(s) of fragment loss. Sequencing revealed that betabodies were cleaved at Lys^317^-Thr^318^ site in domain V (Fig. 2*D*). Factor XI and plasmin are known to cleave β2GP1 at this site as part of thrombus generation (40, 41). This site is significant in that it is directly adjacent to the hydrophobic loop necessary for PS-binding (42). To preserve betabody integrity, we inserted a point mutation K317E which eliminated the cleavage site and preserved PS-binding. The final candidate betabodies that we moved forward with for *in vivo* localization assays were the C and N-terminal versions of KL5 and KL15, all containing the K317E mutation.

### Betabody constructs exclusively bind phosphatidylserine *in vitro*

**Figure 3:**
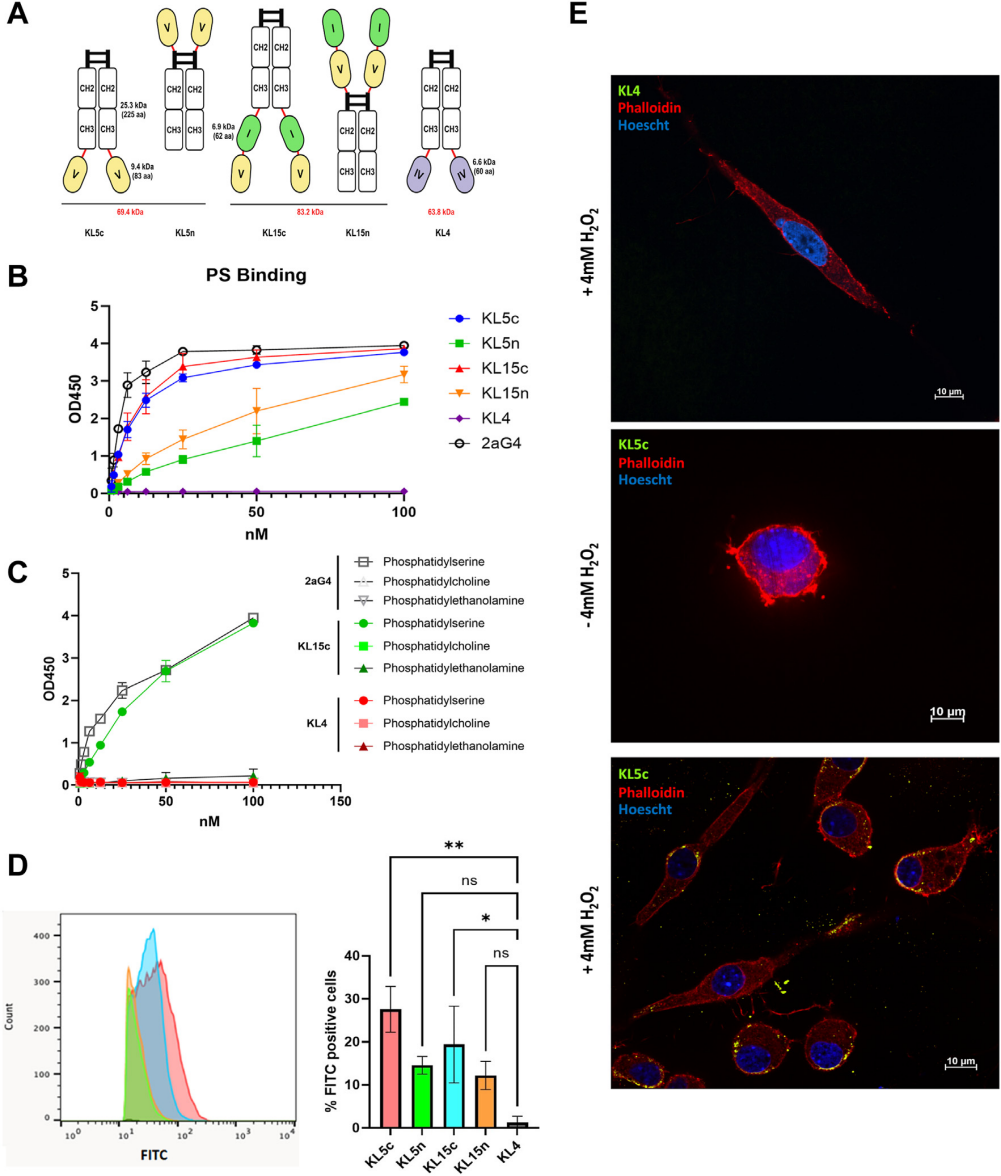
**Betabodies exclusively bind phosphatidylserine *in vitro***. *A*, *schematic of final betabody candidates*. The betabodies were designed by fusing functional PS binding domain of β2GP1 (domain V) to the GGGGS linker and mouse IgG2a Fc domain. KL5c and KL5n contain β2GP1 domain V fused to the C terminus or N terminus of Fc, respectively. KL15c and KL15n contain β2GP1 domain I and V fused to the C terminus or N terminus of Fc, respectively. KL4 mimics KL5c in structure, except it fuses β2GP1 domain IV to Fc. *B*, binding of betabodies to PS *via* ELISA. 2aG4, a PS-targeting antibody, was used as a positive control; KL4 is used as a negative control. *C*, binding to PS, phosphatidylethanolamine (PE), or phosphatidylcholine (PC) *via* ELISA. KL15c and KL4 results presented. *D*, 4T1 cells were treated with 4 mM H_2_O_2_ for 30 min, fixed with 2% paraformaldehyde and then stained with 10 μg/ml of each of the betabodies. Cells were then stained with goat anti-mouse IgG – Alexa Fluor 488 secondary antibody (Invitrogen A-11001; 1:200) and subjected to flow cytometry. *E*, E0771 cells were grown in 3D, treated with 4 mM H_2_O_2_ for 30 min, harvested with trypsin and embedded in 70% collagen with continued treatment with 200 μM H_2_O_2_ in low serum overnight. Cells were fixed with 2% PFA for 1 h and then incubated with 10 μg/ml betabody in PBS. Cells were then stained with secondary antibodies: [*Betabody (Alexa Fluor-488); Phalloidin (Alexa Fluor-564); Nuclei (Hoescht)]*.

The final betabody candidates (Fig. S3) have a similar structure and include a single GGGGS linker between the Fc and β2GP1 domain(s). KL15c and KL5c include β2GP1 domain V (KL5c) or β2GP1 domain I and V (KL15c) connected to the C-terminus of mouse IgG2a Fc. KL5n and KL15n incorporate the same β2GP1 domains fused to the N-terminus of mouse IgG2a Fc (Fig. 3*A*). We also constructed KL4 incorporating β2GP1 domain IV with Fc as a negative control because domain IV does not bind to PS or other lipids. The five betabodies were tested for binding to PS *via* ELISA and compared to the PS-targeting antibody 2aG4 (a mouse IgG2a (26)). As predicted, KL5c and KL15c bound PS comparably to 2aG4, whereas KL5n and KL15n demonstrated diminished binding (Fig. 3*B*). All five betabodies were also tested for binding to phosphatidylethanolamine (PE) and phosphatidylcholine (PC) *via* ELISA. KL5 and KL15 constructs bound PS exclusively (Figs. 3*C* and S4). Betabody binding to externalized PS on cells was tested by flow cytometry of H_2_O_2_-treated 4T1 cells. The results mirrored the PS ELISA. While no betabody matched 2aG4, again the c-terminal constructs bound the highest percentage of cells, with KL5c ultimately having the best cell binding efficacy (Fig. 3*D*). All betabodies also showed limited or no binding to untreated 4T1 cells, demonstrating exclusive PS binding (Fig. S5). With KL5c as the leading candidate, we visually verified the binding of that betabody to H_2_O_2_-treated E0771 cells grown in 3D culture. It is important to note that, because we were staining for binding to lipids, we did not use detergents during the staining process. This resulted in some punctate background staining with the Alexa-488 conjugated secondary antibody throughout the 3D matrix. While the negative control KL4 showed no cell membrane interaction, even with H_2_O_2_ treatment of the cells, KL5c robustly localized to the cell membrane after H_2_O_2_ treatment, in a primarily nuclear-adjacent pattern (Fig. 3*E*). All betabodies were tested for PS-binding on E0771 cells (Fig. S6).

### KL5c selected as the final betabody with robust *in vivo* localization to the TME of 4T1 orthotopic breast tumors

**Figure 4:**
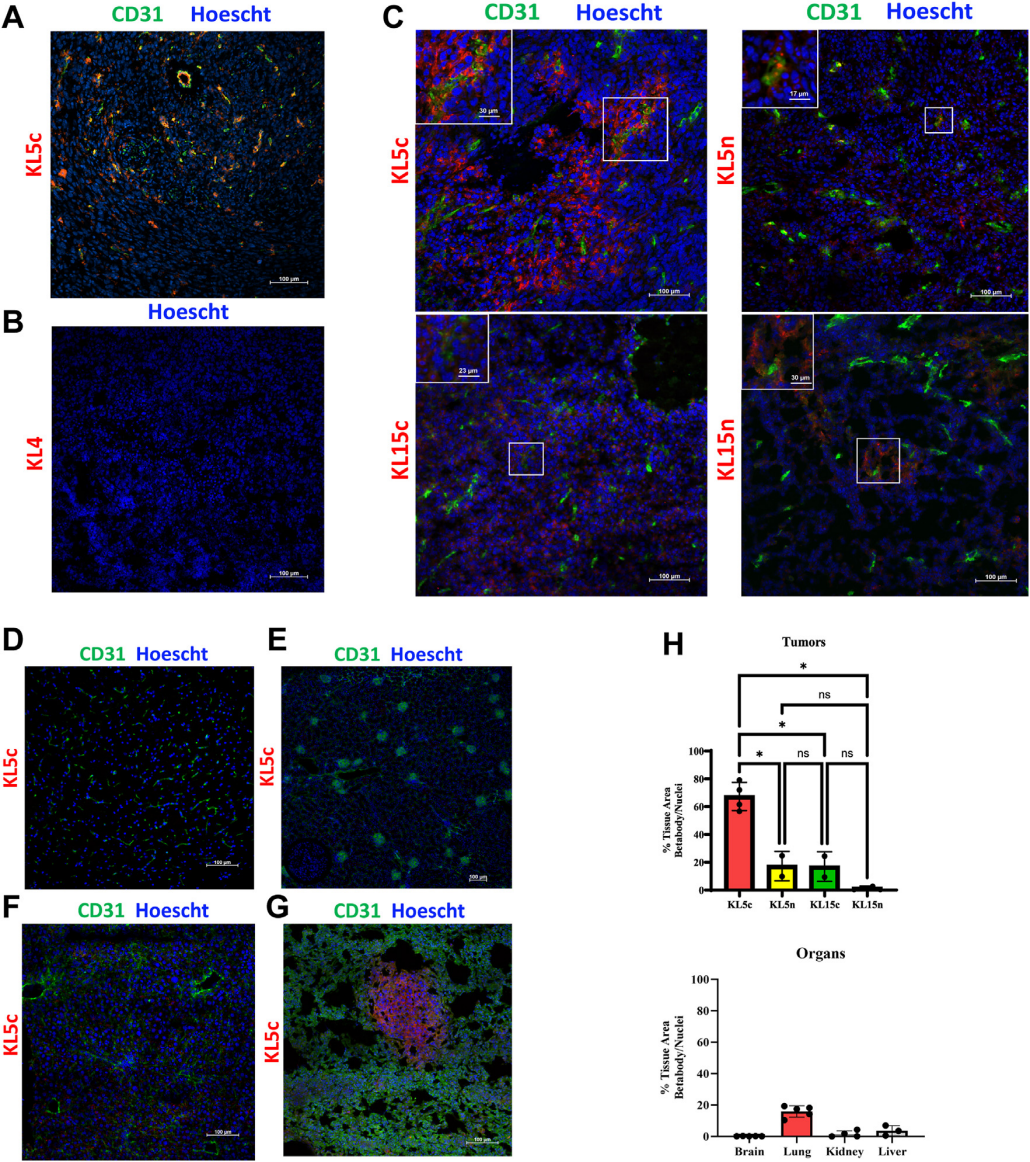
**Betabodies localize to the tumor microenvironment *in vivo***. *A*, *in vivo localization in β2GP1-null mice*. Tumor-bearing Apoh^−/−^ mice (n = 3) were injected i.v. with KL5c (50 μg) that were allowed to circulate for 2 h. Tumors were resected, snap-frozen, and used for IHC to detect KL5c and CD31. Hoescht is used as a counterstain. *B–G*, *In vivo localization studies*. NSG mice bearing 4T1 tumors were injected i.v. with biotinylated KL4 (50 μg) plus the indicated unlabeled betabody (50 μg). Mice were harvested 18 h post-injection, perfused, and organs including tumors collected, snap-frozen, and sectioned. Tumor (b, c), brain (d), kidney (e), liver (f) and lung (g) were stained for KL4 (streptavidin-HRP and Opal 570, *red*) and the betabodies (anti-mouse-HRP and Opal 570, *red*), blood vessels (CD31, anti-goat-HRP, Opal 520, *green*) and nuclei (Hoescht, *blue*). Representative images taken at 20× are shown. *H*, 4× images of all tumors and organ tissues sectioned and stained were analyzed for individual betabody localization using ImageJ. Dots represent biological replicates. Individual betabody signal was compared to the other three using a 2way ANOVA analysis.

To demonstrate that KL5c localizes to externalized PS in a β2GP1-independent manner, localization in KPfC tumors grown in *Apoh*^−/−^ mice was performed. Intravenous injection of an equal amount of betabody into three *Apoh*^−/−^ mice showed that betabodies containing dimerized domain V localized throughout the tumors in the absence of endogenous β2GP1 (Fig. 4*A*) indicating that binding of the betabody to externalized PS is β2GP1-independent.

We next investigated *in vivo* localization of the betabodies in tumor-bearing mice. We injected 4T1 tumors orthotopically into a cohort of NSG mice and let tumors grow for 26 days to allow for metastasis to the lungs to manifest. KL5c (50 μg) or other functional betabodies were mixed with biotinylated KL4 (50 μg), a non-functional betabody, and injected intravenously into 3 mice per each betabody combination. KL4 served as an internal control. Betabodies were allowed to circulate for 18 h before tumors and organs were removed and snap-frozen for IHC. All tumors and normal organs were stained for functional betabody, KL4, and vasculature (CD31). KL4 failed to localize to the tumor microenvironment (Fig. 4*B*) or normal organs. N-terminal and C-terminal betabodies localized to the tumor microenvironment; KL5c demonstrated the most robust localization, with clear binding to the vasculature (inset) and distribution throughout the tumor (Fig. 4*C*). Healthy tissues stained by the same methods as the tumor sections showed no betabody localization (Fig. 4, *D*–*F*) apart from lung tissue (Figs. 4*G* and S10). Strong staining of betabodies in compact, highly nucleated regions of the lungs indicated that betabodies localize to lung metastases (Fig. 4*G*) and the primary tumor (Fig. 4*C*). Identification of the highly nucleated areas as 4T1 metastases was confirmed with IHC staining of a known 4T1 marker, Gpa33 (43) (Fig. S7). All four betabodies demonstrated efficacy *in vivo*, but KL5c illustrated its superiority based on more widespread dispersion throughout the tumor, quantified by ImageJ and analyzed for significance using a 2Way ANOVA (Figs. 4*H* and S8). Additionally, KL5c was tested in competition with the well-characterized PS-binder Annexin V and was able to effectively compete for PS binding (Fig. S11). Therefore, KL5c is the final betabody construct that will be used in future experiments.

## Discussion

β2GP1 has been studied in the context of APS, thrombosis, and pregnancy loss (44, 45, 46). However, outside of its contribution to PS-targeting antibodies, β2GP1 has not been utilized as a tool to target the TME. Though we have shown previously *in vitro* that β2GP1 is a required cofactor for the interaction of PS-targeting antibodies with PS (24), here we demonstrate that it is unequivocally required for the localization of PS-targeting antibodies (*e.g.*, bavituximab) to externalized PS *in vivo*. Utilizing this relationship, we designed a construct that binds PS directly and contains an Fc domain. Multiple permutations of β2GP1 domains and Fc orientations were tested for efficacy, however the simplest design was the most effective. The combination of Fc fused to domain V, the sole PS-binding domain of β2GP1, with a single linker is the superior iteration of betabody for PS localization to the TME.

We developed the Apoh^−/−^ mouse as a negative control to demonstrate the indispensable relationship between PS and β2GP1 for PS-targeting. Because domain V is the only domain of β2GP1 that binds to PS, an alternative strategy could have been to produce a mouse that only expressed β2GP1 domains I-IV. While, this strategy would definitively isolate the PS-β2GP1 relationship to domain V *in vivo*, the requirement of domain V for PS interaction has already been demonstrated (47). Eliminating β2GP1 in its entirety was an efficient strategy given the mice were available from UC Davis KOMP Repository Knockout Mouse Project. In addition, β2GP1 domains are associated with interaction with other proteins. For example, sequences of residues within the primary structure of domain III of β2GP1 mimic peptides from infectious bacteria and viruses; these peptides can give rise to antibodies associated with an APS phenotype (48). In addition, domain V serves to hold β2GP1 in an inactive “Q” confirmation in circulation through interaction with domain I, masking cryptic epitopes on the domain I that are recognized by APS antibodies (32). Eliminating domain V alone might make endogenous β2GP1 more immunogenic. Our development of KL4, utilizing non-PS-binding domain IV, demonstrates specific binding of domain V of β2GP1 to externalized PS.

Though encouraging that *in vivo* experiments have demonstrated that betabodies function as anticipated in tumor-bearing animals, the question about the specificity of these constructs for the TME remains to be answered. PS is exposed regularly in multiple healthy tissues. For example, the epithelium of the gastrointestinal tract, cells of the thymus and bone marrow, and germ cells in the testes undergo apoptosis regularly (49), yet we have not detected betabody localization to these tissues *in vivo*. Additionally, there are other cells that are non-apoptotic and not associated with tumors that externalize PS as a signaling mechanism, including differentiating monocytes and certain populations of T cells (50, 51). These cells might be bound and affected by betabodies (52). However, in our experiments, betabodies do not appear to be accumulating in these tissues or being cleared by immune cells. Instead, betabodies localize strongly to the TME. Similarly, duramycin, a highly specific phosphatidylethanolamine (PE)-binding peptide, has been used for imaging of the tumor vasculature; PE is also externalized on apoptotic cells, and duramycin localizes specifically to the TME (53). The reason for this is not yet clear.

An additional concern is that the circulation of perpetually dimerized domains of β2GP1 may interfere with the function of endogenous β2GP1. While β2GP1 must have a biological function as it is known to be maintained at a high concentration, approximately 200 μg/ml, in serum and is highly conserved throughout the mammalian class (30), the specifics as to what the function of β2GP1 is have not been definitively identified. It is well known that β2GP1 is the primary antigen for pathogenic antibodies in APS (45, 46); however, the mechanisms by which β2GP1 is involved in the pathology are unclear. Indeed, humans and mice that lack β2GP1 are viable and healthy (54).

One of the advantages of betabodies over PS-targeting antibodies is their size, but the inclusion of the Fc domain increases the size. The Fc domain is highly useful as a tag for purification and for attachment of labels for imaging purposes; however, generation of a cleavable Fc domain and its removal would permit more rapid and efficient penetration into solid tumors and allow for more rapid clearance of excess betabody from circulation. This could be a favorable characteristic if betabodies had cytotoxic drugs conjugated to them in the manner of an antibody drug conjugate (ADC) or if betabodies were used to image the TME. Indeed, duramycin-conjugates, which are cleared rapidly (within 4 min rats), have been used to non-invasively image the TME (53, 55).

Several labs, including our own, have developed PS-targeting agents that have shown efficacy in preclinical models and in human patients (25, 26, 27, 52, 56, 57, 58). For example, Bavituximab has advanced into clinical trials (NCT03519997, NCT04150900, NCT03139916). Some of these constructs exhibit internalization which could contribute to off-target effects, especially in immune cells that externalize PS, though neither PS-targeting antibodies nor betabodies have been shown to internalize when bound to PS. The mechanism of action of PS-targeting antibodies appears to be driven on the outside of tumor cells (20). Beyond just PS-targeting antibodies, other PS-specific agents have shown efficacy against multiple tumor types. SapC-DOPS nanovesicles have been developed that directly target tumor cells by binding to externalized phosphatidylserine and inducing caspase-mediated apoptosis (59, 60, 61). This approach bypasses the immune response, targeting tumor cells directly. Another group has also developed a fusion protein targeting PS, Fc-Syt1, by which they deliver a cytotoxic drug through internalization of the construct (52). We have not seen evidence of internalization of PS-targeting antibodies or betabodies by tumor cells, and, in fact, the bivalent Fc-Syt1 protein, analogous to betabodies, exhibited limited internalization compared to their tetravalent agent (52).

We envision the use of betabodies such as KL5c as a delivery agent for therapeutic payloads to the TME, where payloads are released from the betabody through extracellular proteases (62). This would allow for an additional layer of specificity for drug targeting. This is the next step for betabodies, the next generation of PS-targeting agents.

## Experimental procedures

### Betabody expression constructs

All betabody coding sequences excluding KL15c and KL15n were cloned into pEE12.4 plasmids (Lonza Biologics; Tables 1 and 2) using XmaI and EcoRI restriction sites and primers (Table S1). KL15c and KL15n were ordered from VectorBuilder.

**Table 2:** Final betabody constructs

Betabody Construct	2-glycoprotein-1 domains	N- or C-terminal fusion	Number of linkers	Mutations
KL5c	5	C	1	K317E
KL5n	5	N	1	K317E
KL15c	1,5	C	1	K317E
KL15n	1,5	N	1	K317E
KL4^a^	4	C	1	-

a Negative Control.

### Cell lines

All breast cancer cell lines (4T1, E0771) were obtained from ATCC. Pancreatic ductal adenocarcinoma cell line KPfC were isolated from a *Kras*^*LSL−G12D/+*^*Trp53*^*fl/fl*^*Pd x 1*^*Cre/+*^ GEMM.

### Betabody production and purification

Betabody were produced and purified as previously described (63). Briefly, plasmids for each construct were transiently transfected into Expi293F suspension cells (Gibco A14528). After 5 days, the supernatant was harvested by ultracentrifugation and filtration. Supernatants were diluted by 50% with binding buffer (50 mM Tris, 50 mM NaCl, pH 8.2) and run over a protein A column (Prosep-vA High-Capacity Affinity Chromatography Media, Millipore C175805). Betabodies were eluted using acetate buffer (100 mM sodium acetate, pH 2.8) and neutralized with 10% volume of 1 M Tris pH 9.0. Betabodies were purified using Cytiva AKTA pure chromatography system.

### Generation of **β**2GP1 null C57Bl/6 mice

Two heterozygous/hemizygous embryonic stem cell clones derived from a C57Bl/6NTac background were procured from the UC Davis KOMP Repository Knockout Mouse Project and microinjected into blastocyst embryos. Both clones contained a ZEN-UB1 lacZ reporter/neo selection cassette (*Apoh*^*tm1(KOMP)Vlcg*^) inserted into the *Apoh* locus on chromosome 11 *via* homologous recombination between location 108,395,347 and location 108,414,365, resulting in a complete deletion of the gene. Heterozygous knockouts of the Apoh gene were backcrossed with C57Bl/6 mice for six generations, before being crossed with Cre^+^ C57Bl/6 mice (B6.C-Tg(CMV-cre)1Cgn/J; IMSR_JAX:006054) to remove the Neo cassette (Fig. 1*B*). *Apoh*^+/−^ *Cre*^+^ offspring were backcrossed with WT C57Bl/6 mice for a final heterozygous N7 generation lacking Cre. The genetic background of the N7 offspring was tested by the DartMouse Lab at the Dartmouth Geisel School of Medicine and confirmed to be 99% C57Bl/6 background. N7 *Apoh*^+/−^ *Cre*^-^ mice were crossed to produce the first generation of *Apoh*^−/−^ mice. Homozygous Apoh knockout mice were verified by genotyping for the WT gene and LacZ cassette using primers 26,27 and 24,25 respectively (Table S1), RNA and protein fractions extracted from mouse livers were tested by qPCR using primers 21,22 (Table S1), and Western blot (Proteintech 11892-1-AP) for β2GP1 RNA and protein expression, respectively. *Apoh−/−* mice were generated as described in the Results (Fig. 1*B*). All animals were housed in a pathogen-free facility with access to food and water ad libitum. Experiments were performed under protocols approved by the institutional animal care and use committee at the University of Texas Southwestern Medical Center in Dallas.

### 1N11 *in vivo* localization

*Apoh*^−/−^ C57Bl/6 mice (n = 6) and WT C57Bl/6 mice (n = 3) were injected with 1 million KPfC pancreatic cancer cells (100 μl PBS) subcutaneously in the right flank. Tumors were allowed to grow to ∼500 mm^3^. *Apoh*^−/−^ mice were injected with 50 μg 1N11 (n = 3) or 50 μg 1N11 preincubated with 35 μg purified β2GP1 (1N11: β2GP1 molar ratio of 1:2) (n = 3) *via* the tail vein. The C57Bl/6 group was injected with 50 μg 1N11 (note 1N11 a human PS-targeting antibody binds to mouse and human β2GP1 (36)). The antibody was allowed to circulate for 2 h at which point the mice were euthanized and perfused with PBS. Tumors were resected, snap frozen, and sectioned. Localization of the injected antibody was determined by immunohistochemistry (IHC). Frozen slides with 8 μm sections of tumors were fixed with ice-cold 4% PFA for 15 min. Endogenous peroxidases were blocked with 0.1% H2O2 in PBS for 5 min; tissue was then blocked for 30 min 1N11 were identified with anti-human IgG HRP (1:100; Jackson Immunoresearch 109–035–008) followed by Opal 570 (Akoya Biosciences FP1488001KT). Antibodies were stripped with antibody elution buffer (Glycine-SDS pH 2) (42) at 50ºC for 30 min; vasculature was then stained with anti-CD31 (R&D Systems AF3628) followed by anti-goat IgG HRP (Jackson Immunoresearch 705–035–147) and Opal 520 (Akoya Biosciences FP1487001KT). Nuclei were counterstained with Hoescht 33,342 (Invitrogen H1399).

### Betabody *in vivo* localization

Four groups of Nod.Cg-*Prkdcscid Il2rgtm1Wjl/SzJ* (NSG) mice (n = 3) were injected with 100,000 4T1 cells (100 μl PBS) into the fourth mammary fat pad. Primary tumors were allowed to grow for 3 weeks to allow for metastasis to the lungs. Each group was treated with a combination of 50 μg of one of the functional betabodies (KL5c, KL5n, KL15c, or KL15n) and 50 μg of a biotinylated negative control betabody (KL4) *via* tail vein injection. Betabodies were allowed to circulate for 18 h; mice were then humanely euthanized and perfused with PBS before resection of tumors and organs. Resected tissues were bisected, with half of each preserved in formalin and half snap frozen in liquid nitrogen for betabody localization staining. IHC staining was performed using the same methods as for the 1N11 localization except that: functional betabodies were identified with anti-mouse IgG HRP followed by Opal 570 (red) and KL4-biotin was identified by HRP-streptavidin followed by Opal 570 on a separate tissue section. Vasculature was stained with anti-CD31 followed by anti-goat HRP and Opal 520 (green). Nuclei were counterstained with Hoescht (blue).

### Anti-PS ELISA

Phosphatidylserine (Avanti Polar Lipids, Inc. 840032C-25 mg) was dissolved in n-hexane to a concentration of 10 μg/ml. 50 μl of the solution was added to each well of a 96-well Immulon 1B microtiter plate (#4466966), and the solvent was allowed to evaporate. Plates were blocked for 1 h at room temperature with 5% nonfat milk in PBS (w/v). Betabodies (100 nM) were subjected to serial 2-fold dilution in the blocking buffer (100 μl per well), and plates were then incubated for 1 h at 37 °C. Betabodies were detected with HRP-conjugated anti-mouse (Fcγ specific) (1:3000) for 1 h at 37 °C. Plates were washed four times with 200 μl of PBS per well after betabody and secondary antibody incubation. 2aG4, a mouse PS-targeting antibody was used as a positive control (18). Secondary reagents were detected by chromogenic substrate TMB and stopped with equal volume of 10% HCL. Plates were read at 450 nm using a microplate reader. Negative controls included incubation of non-PS binding betabody KL4 and incubation of betabodies in uncoated wells.

### Flow cytometry

4T1 cells were treated with 4 mM H_2_O_2_ in serum-free DMEM for 30 min. Cells were washed with cold PBS, trypsinized, and washed three times with flow buffer (Dulbecco's Phosphate Buffered Saline + 0.5% BSA). Cells were fixed with 2% PFA and then betabody was added directly to the cell suspension, final concentration of 10 μg/ml, for 1 h on ice. After 1 h, cells were spun down and washed three times with buffer. Cells were suspended in 500 μl of anti-mouse Alexa Fluor-488 (1:200) and incubated on ice in the dark for 30 minutes. Controls included unstained cells and AnnexinV-FITC (positive control, 1:100). Cells were spun down and washed three times with buffer and analyzed on a FACSCaliber machine in the UTSW Flow Cytometry Facility.

### 3D immunofluorescence (IF) staining of E0771 cells

Experiments in three-dimensional (3D) culture were performed as described previously (64). Briefly, Cultrex UltiMatrix Reduced Growth Factor Basement Membrane Extract (10–12 mg/ml stock concentration, R&D Systems, BME001–05) and Rat Tail Collagen I (Corning, catalog no. 354236) were used for organotypic culture experiments. 3D assays were plated using a 30% Cultrex/70% Collagen I matrix (3–3.6 mg/ml Cultrex and 2.1 mg/ml Collagen I). Cells were treated with 4 mM H_2_O_2_ for 30 min and then embedded in the Cultrex/Collagen matrix. After setting overnight, cells were fixed with 2% PFA for 1 h and then blocked with 2% bovine serum albumin (BSA) in PBS and incubated with 10 μg/ml betabody for 2 h at 37 °C. Cells were then stained with anti-mouse Alexa Fluor-488 (1:500), AlexaFluor-568 Phalloidin (1:500), and Hoescht (1:200) (Invitrogen, 33342). Images were taken using the spinning disk confocal Nikon CSU-W1 with SoRa in the UTSW Quantitative Light Microscopy Core.

## Data availability

All data relevant to betabody characterization is contained within the body of the manuscript and supporting information.

## Supporting information

This article references supporting figures contained within the supporting information.

## Conflict of interest

The authors declare the following financial interests/personal relationships which may be considered as potential competing interests:

The authors declare that they have no conflicts of interest. A patent (US 8,956,616 B2) on betabodies has been issued to the University of Texas System, the authors of this manuscript are not associated with the patent.
