# Supplementary material for: Development of betabodies: The next generation of phosphatidylserine targeting agents
Source: J Biol Chem. 2024 Aug 17;300(9):107681. doi: 10.1016/j.jbc.2024.107681 (PMC11416255; doi:10.1016/j.jbc.2024.107681)
Supplement: Supporting Information [file mmc1.docx]

Development of betabodies: the next generation of phosphatidylserine targeting agents

**Natalie Phinney^1,2,3^, Jason E. Toombs^1,2^, Xianming Huang^2,4,#^, Rolf A. Brekken^1,2,3,4,5*^**

# ^1^Department of Surgery, ^2^Hamon Center for Therapeutic Oncology Research, ^3^Cancer Biology Graduate Program, ^4^Department of Pharmacology, ^5^Harold C. Simmons Comprehensive Cancer Center, UT Southwestern Medical Center, 6000 Harry Hines Blvd., Dallas, TX, 75390-8593, USA.

^#^Current address: Bio-Thera Solutions, Ltd., Floor 5, Building A6, 11 Kai-Yuan Blvd, Huangpu District, Guangzhou, China, 510530

**Supporting Information:**

- **Table S1.** Primer sequences.
- **Figure S1.** Modification of β2-Glycoprotein 1 domains disrupts binding to PS.
- **Figure S2.** Serum proteases are responsible for betabody degradation and loss of PS binding.
- **Figure S3.** Betabody constructs.
- **Figure S4.** Betabodies bind exclusively to phosphatidylserine.
- **Figure S5.** H_2_O_2_ treatment of 4T1 cells increases phosphatidylserine externalization and betabody binding to the cell membrane.
- **Figure S6.** All betabodies bind to H_2_O_2_ treated cells in vitro to varying degrees.
- **Figure S7.** Lung metastasis markers.
- **Figure S8.** KL5c demonstrates the most robust in vivo localization throughout the body of the whole 4T1 tumor.
- **Figure S9.** KL4 shows no localization to the TME.
- **Figure S10.** Organs stained for betabody showed no localization outside of 4T1 metastases in the lungs.
- **Figure S11.** Competition assay.

**Table S1.** Primer sequences.

|  | Primers | Sequence |
| --- | --- | --- |
| 1 | **KL4_cloning_F** | ACTCCCGGGGGAGGTGGCTCGGTAAAATGTCCCTTCC |
| 2 | **KL4_cloning_R** | GATCAATGAATTCATTACTCTCTACAGGTCGGC |
| 3 | **DL15_F** | ACGAGCACAGTTCTCTGGCTTTC |
| 4 | **DL15_R** | CGAAGCACGAGGGAATCTCGATAG |
| 5 | **SL15_F** | CGGAGCACAGTTCTCTGGCTTT |
| 6 | **SL15_R** | AGAAGCACGAGGGAATCTCGATAG |
| 7 | **AL15_F** | CGGAGCACAGTTCTCTGGCTTT |
| 8 | **AL15_R** | CGAAGCACGAGGGAATCTCGATAG |
| 9 | **D1_dg** | AACACCCTGAGATGTGTCCCCGGAGGTG |
| 10 | **D2_dg** | GATATTCCTGCTTGTGCTCGCGGAGGTG |
| 11 | **D5_dg** | GAGGCTCGTCTTGCAAACTCCCCGTTAAG |
| 12 | **G4S_LINKER_F** | GTGACTCCCGGGGGAGGTGGCTCGGGACGGATCTGTCCGAAGCCG |
| 13 | **(G4S)3_LINKER_F** | GTGACTCCCGGGGGAGGTGGCTCGGGCGGAGGTGGGTCGGGTGGCGGCGGATCAGGACGGATCTGTCCGAAGCCG |
| 14 | **(G4S)5_LINKER_F** | GTGACTCCCGGGGGAGGTGGCTCGGGCGGAGGTGGGTCGGGTGGCGGCGGATCAGGCGGAGGTGGGTCGGGTGGCGGCGGATCAGGACGGATCTGTCCGAAGCCG |
| 15 | **LINKER_R** | CAATGAATTCATTAGCACGGTGTCAGTTCTGATGC |
| 16 | **Fc3-5_F** | GACTCCCGGGTCACCTGCCCGCCACCACCAG |
| 17 | **Fc3-5_linker_F** | GACTCCCGGGGGTGGAGGCGGATCGATCACCTGCCCGCCACCACCAG |
| 18 | **Fc3-5_R** | GATCAATGAATTCGACTTCAGC |
| 19 | **Fc2-5_F** | CCCGGTTTTACCCGGAGTCCGAG |
| 20 | **Fc2-5_R** | AGAGTATGTCCTTTCGCTGGAATC |
| 21 | **B2GP1_F** | GACAGCTGGAACACCAGTGA |
| 22 | **B2GP1_R** | CCGTCTTGGTACATTCCGCT |
| 23 | **Reg-NeoF** | GCAGCCTCTGTTCCACATACACTTCA |
| 24 | **Reg-LacF** | ACTTGCTTTAAAAAACCTCCCACA |
| 25 | **Reg-Apoh-R** | AAACAGAACAAGGGAAGGGCAGAGG |
| 26 | **Reg-Apoh-wtF** | CTCTTGCTTCTCTCTCTTGCTTCGC |
| 27 | **Reg-Apoh-wtR** | CCTTCCTTCCTTCCTTCCTTCCTCC |

**Figure S1. Modification of β2-Glycoprotein 1 domains disrupts binding to PS.**  A. One Balb/c mouse was injected with 20 µg of ^125^I-labeled FLB; after ten minutes, indicated organs were weighed, and the radioactivity of each organ was counted by γ-counter. B. FLB was first chemically de-glycosylated; 100 units of PNGase F was added to 900 µg of FLB in PBS. Samples were incubated at 37 ºC for the indicated time points and then tested for activity using PS ELISA. Additionally, FLB, F3LB, and F5LB were each genetically de-glycosylated by using site-directed mutagenesis and tested for activity by PS ELISA. C. KL15 was genetically mutated using site-directed mutagenesis at K308 to remove the ApoER2 binding site on Domain V; K was changed to A, D or S and tested for activity using PS ELISA. D. Irradiated Daudi cells were treated with K308 mutants and analyzed for PS binding using flow cytometry.


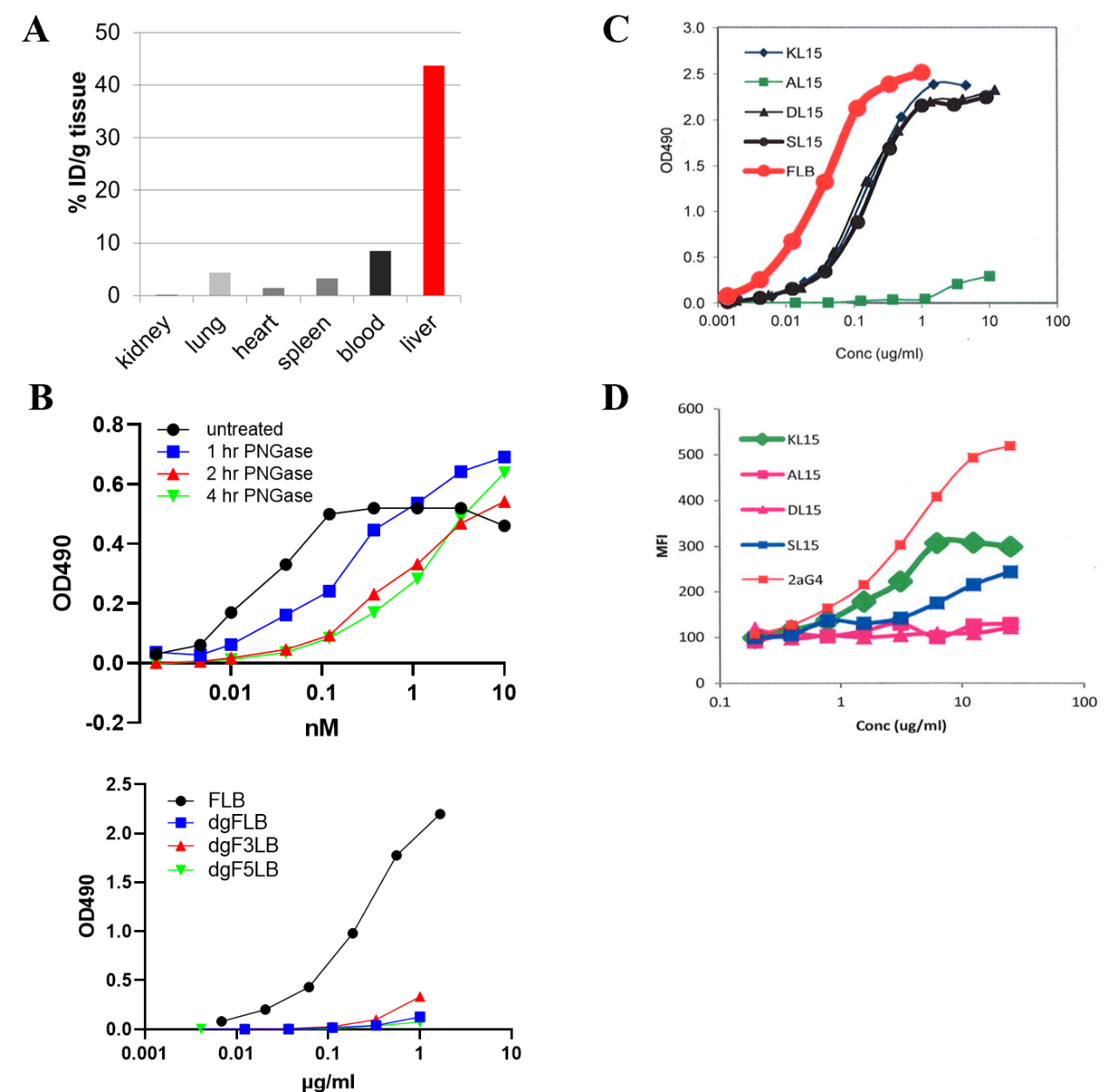


**Figure S2. Serum proteases are responsible for betabody degradation and loss of PS binding.**  A. 15 µg of ^125^I-labeled KL15 and SL15 was injected i.v. Blood was collected at indicated time points, and betabody concentration was measured by γ-counter (n=5). B. SCID mice were injected i.v. with 100-200 µg of betabody; at indicated timepoints, animals were sacrificed, and blood was harvested. Betabody concentration was measured by capture ELISA and activity determined by PS ELISA. Each curve represents data from a single mouse. Controls are purified betabodies. Curves shown indicate betabody activity. C. Purified KL15c was added to freshly collected plasma or serum from naïve mice. Samples were then incubated at 37 ºC; at various time points, samples were analyzed by PS ELISA for PS-binding activity. Results indicate that KL15c is stable in plasma but relatively unstable in serum over time. D. 200 µg to 1 mg of KL15c or KL5c were injected into SCID mice; after 24 hours, mice were injected with heparin then sacrificed, and blood was harvested. Betabodies were purified from the plasma by Protein A column and then analyzed for cleavage by SDS gel under reducing conditions.


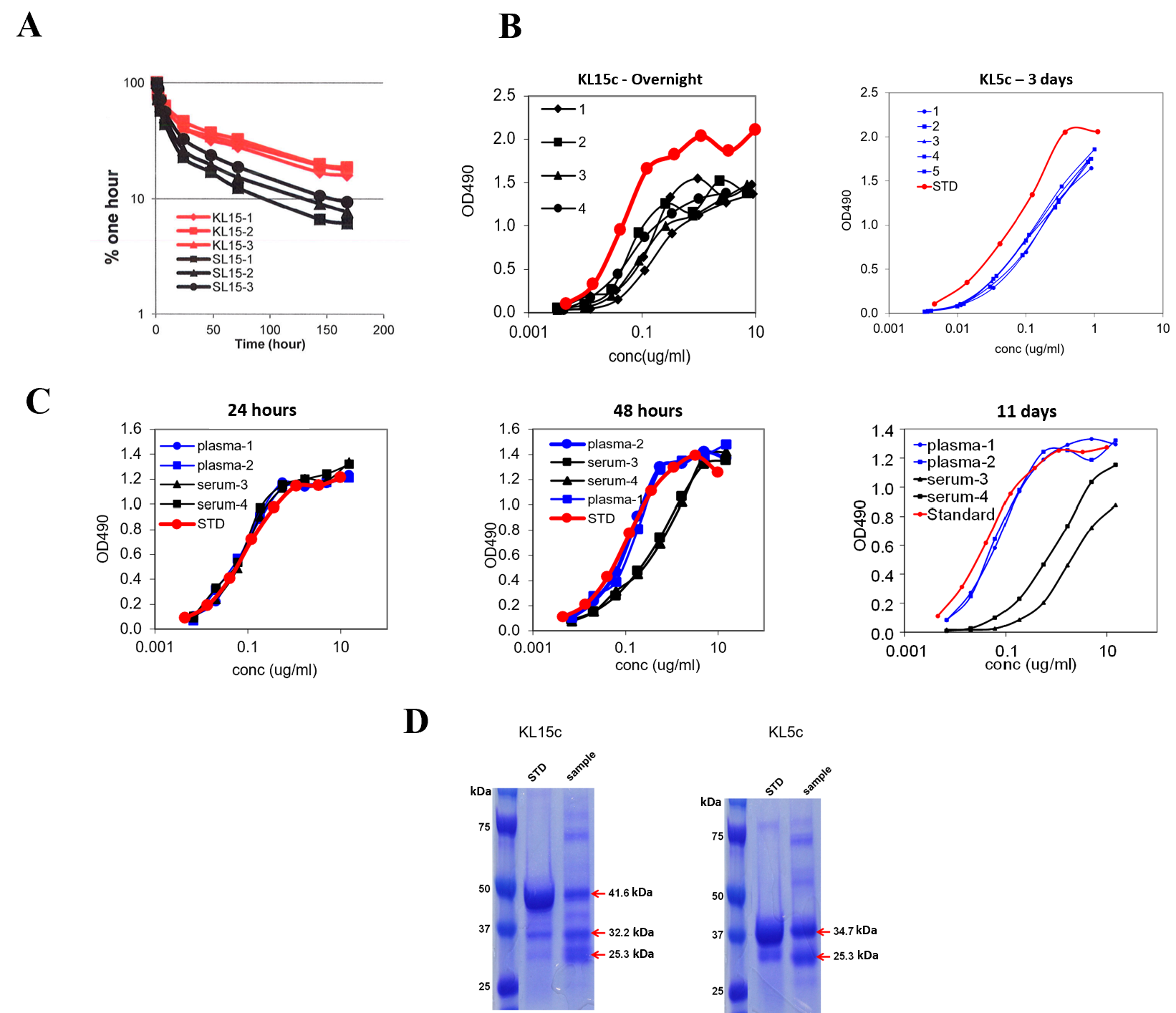


**Figure S3. Betabody constructs**. The indicated constructs were verified to be the correct size after purification by SDS PAGE gel under reducing conditions. Gels were stained with Coomassie dye to visualize the purified protein. Below each gel is the schematic of the relevant betabody, indicating the structure and molecular weight of each part of the construct.


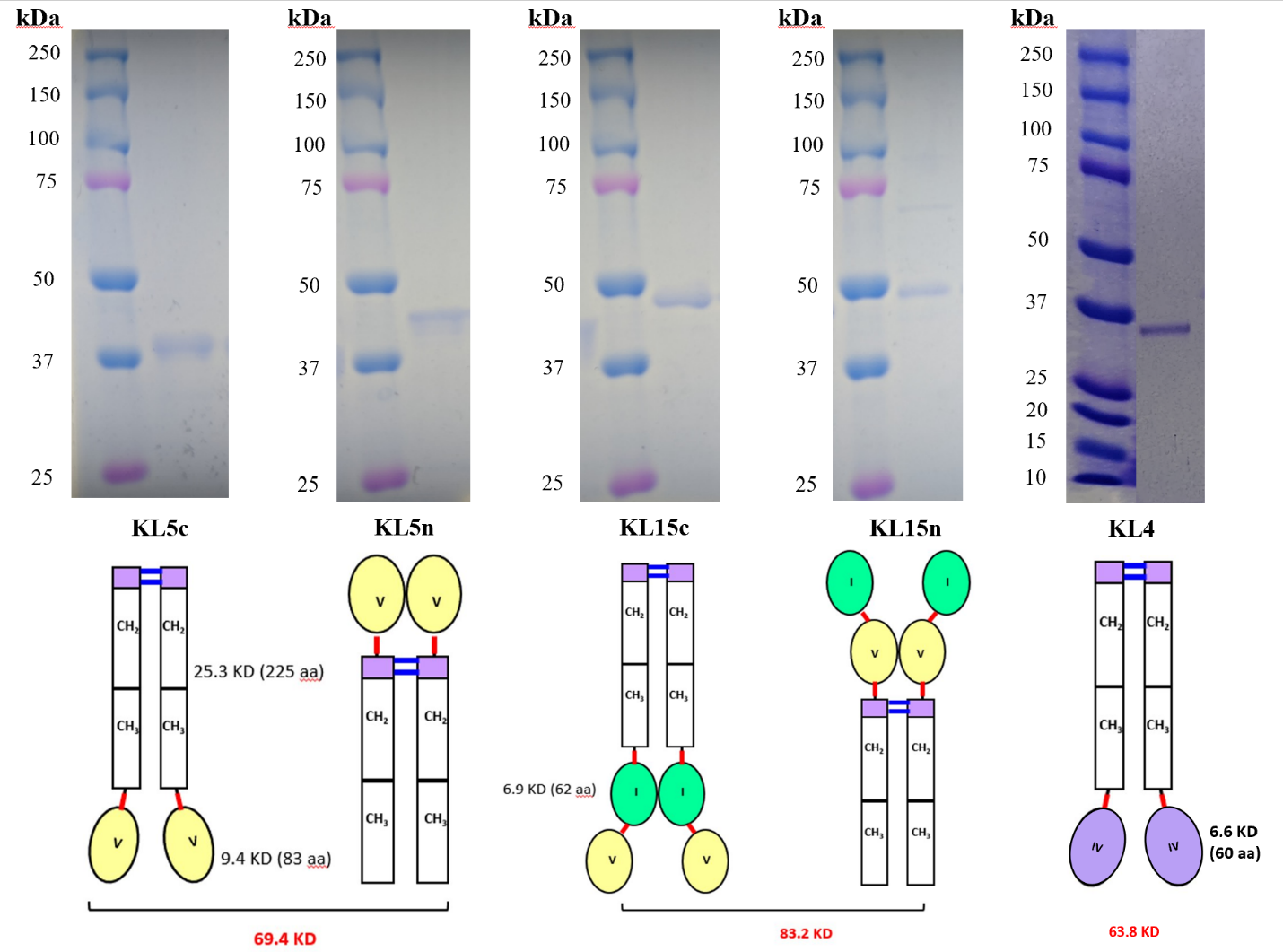


**Figure S4. Betabodies bind exclusively to phosphatidylserine.** Betabodies were tested for PS specificity using a similar ELISA assay to the PS ELISA. Wells were coated with the same concentration of PS, phosphatidylethanolamine (PE), or phosphatidylcholine (PC) and then incubated with betabody; betabodies were all probed with anti-mouse Fcγ specific secondary antibody.


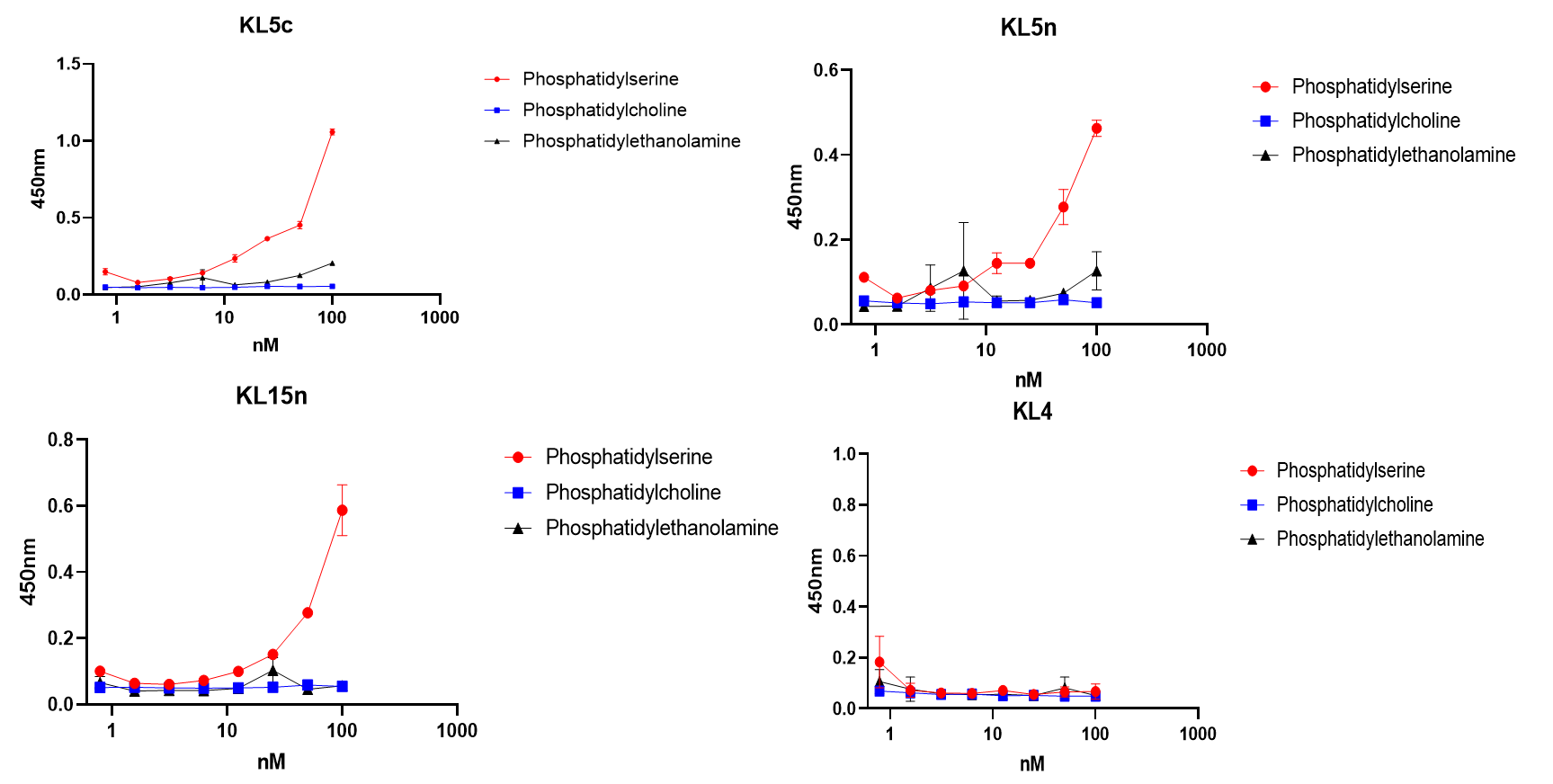


**Figure S5. H_2_O_2_ treatment of 4T1 cells increases phosphatidylserine externalization and betabody binding to the cell membrane.** Each of the five betabodies was tested for binding to both untreated 4T1 cells and cells treated with 4 mM H_2_O_2_ and measured with flow cytometry. Treated cells were preincubated with 4 mM H_2_O_2_ for 30 minutes prior to incubation with betabody and staining with anti-mouse Alexa Fluor 488 (1:200).


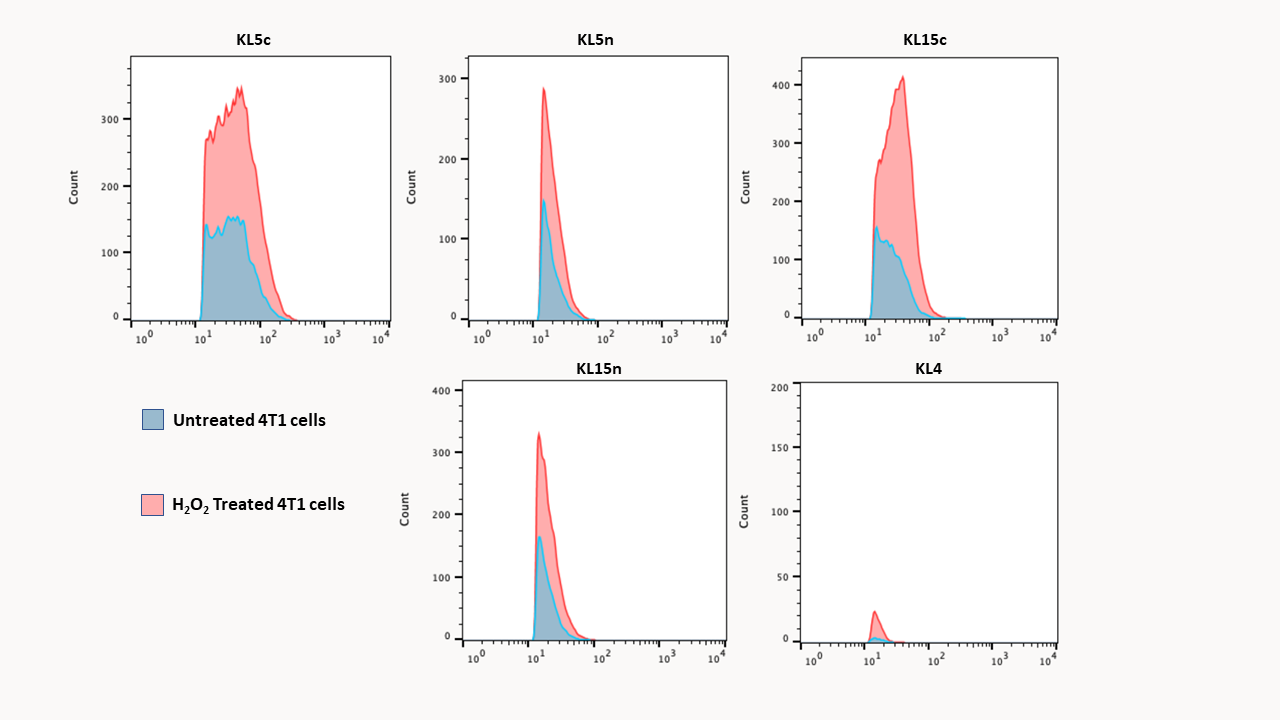


**Figure S6. All betabodies bind to H_2_O_2_ treated cells in vitro to varying degrees.** A-F. In vitro binding was tested on multiple cell lines with all five betabodies, with and without H_2_O_2_ treatment. Cells were plated on glass coverslips coated with fibronectin; after overnight incubation, cells were treated with 200 µM H_2_O_2_ for one hour to induce PS externalization. Cells were fixed with 0.25% gluteraldehyde and stained with betabodies, biotinylated anti-mouse Fcγ-specific secondary, and Cy3-streptavidin. Nuclei were stained with Hoescht. [KL5c (A.); KL5n (B.); KL15c (C.); KL15n (D.); KL4 (E.); KL5c with no H_2_O_2_ treatment] G. Cells were quantified for betabody localization to cells using ImageJ analysis of 10x images.


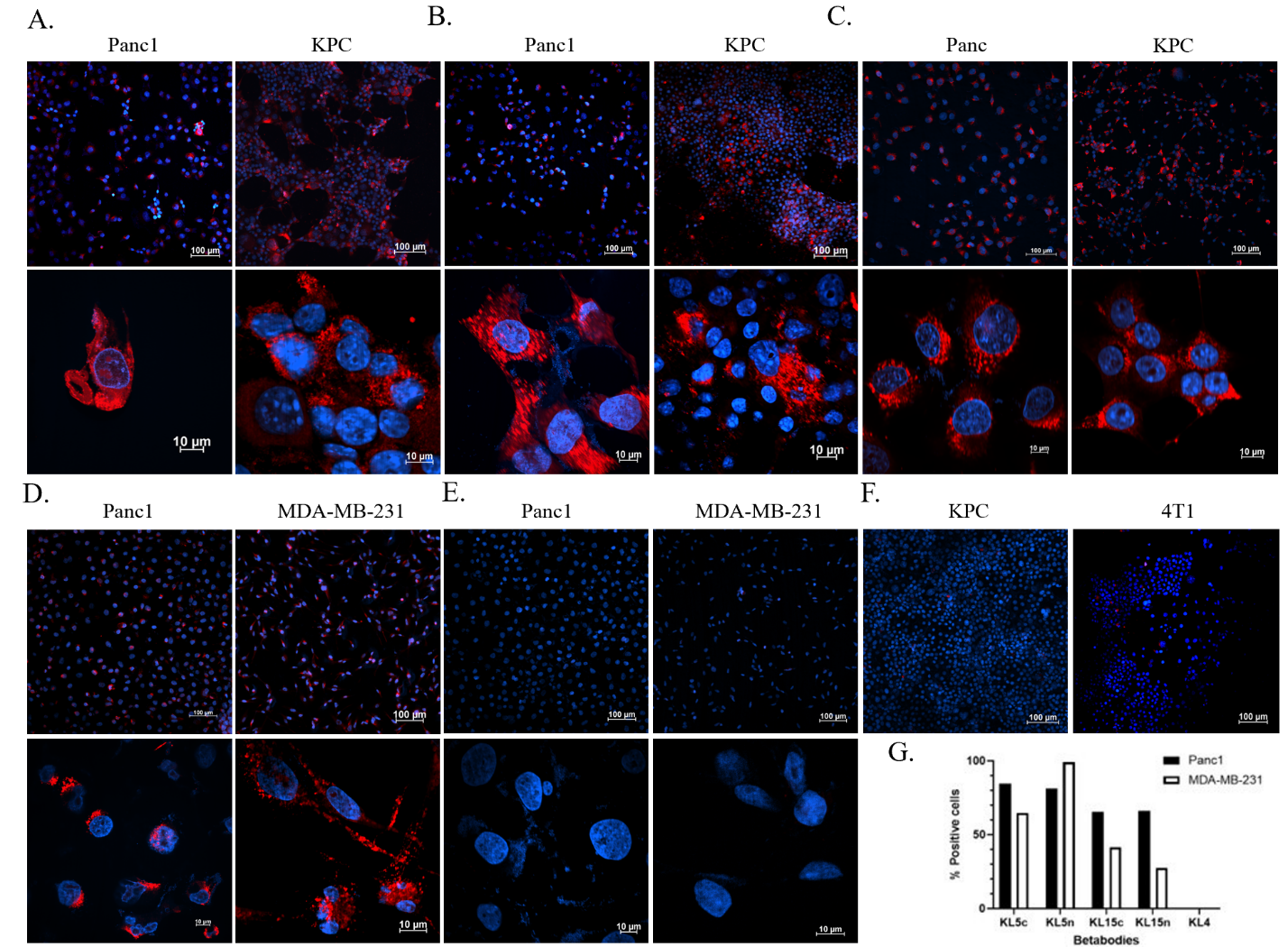


**Figure S7. Lung metastasis markers.** IHC of A. Lungs from a non-tumor bearing mouse B. 4T1 primary tumor C. Lungs with 4T1 metastases from a mouse that had been tail vein injected with KL5c. Tissues were stained with 4T1 marker Gpa33 (DAB) to verify 4T1 metastases in the lungs of betabody-treated mice in the in vivo localization experiments.


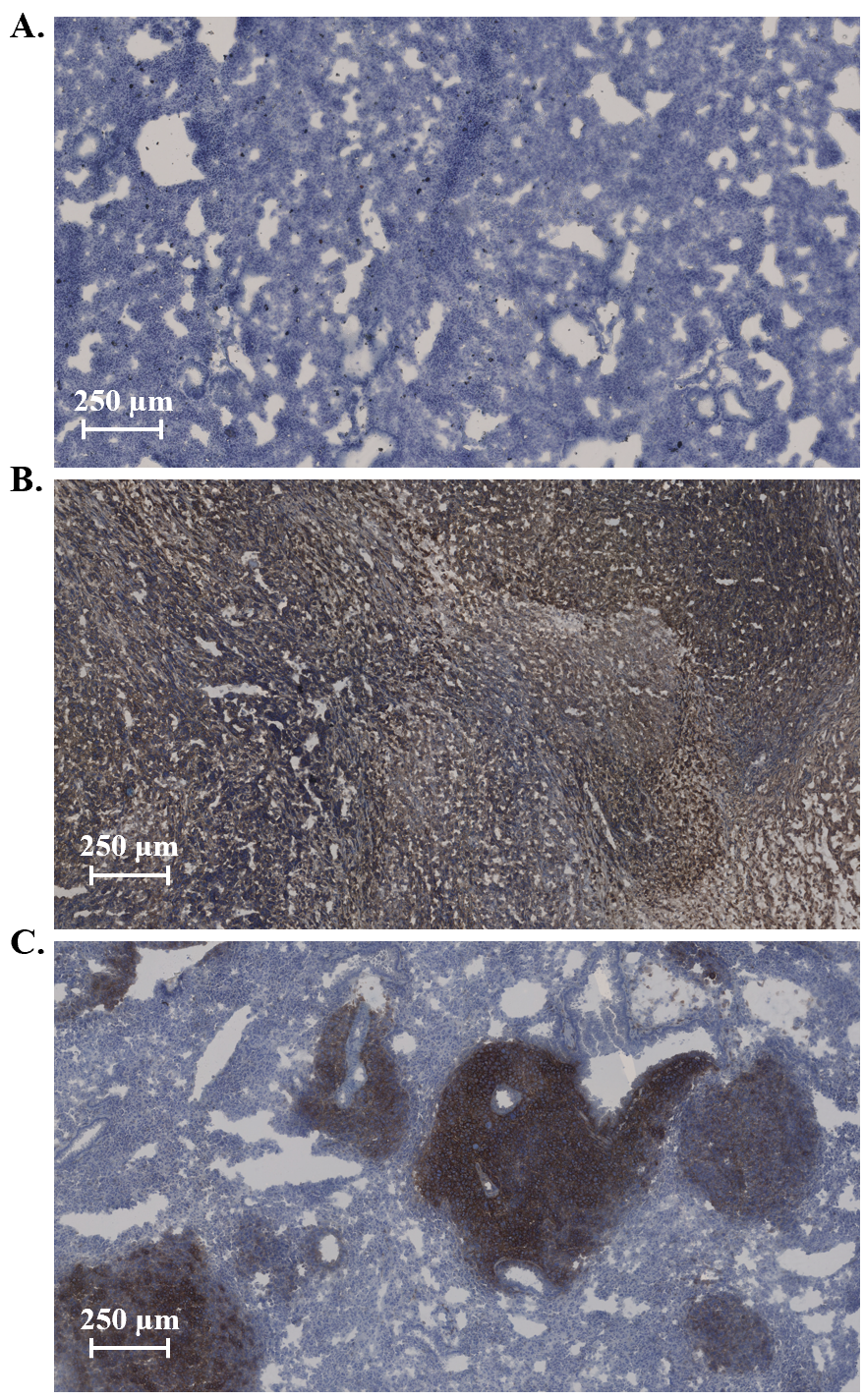


**Figure S8. KL5c demonstrates the most robust in vivo localization throughout the body of the whole 4T1 tumor.** Betabodies were stained for in 4x sections using HRP-anti-Ms Fcg specific and Opal 690. Each image is a different tumor.


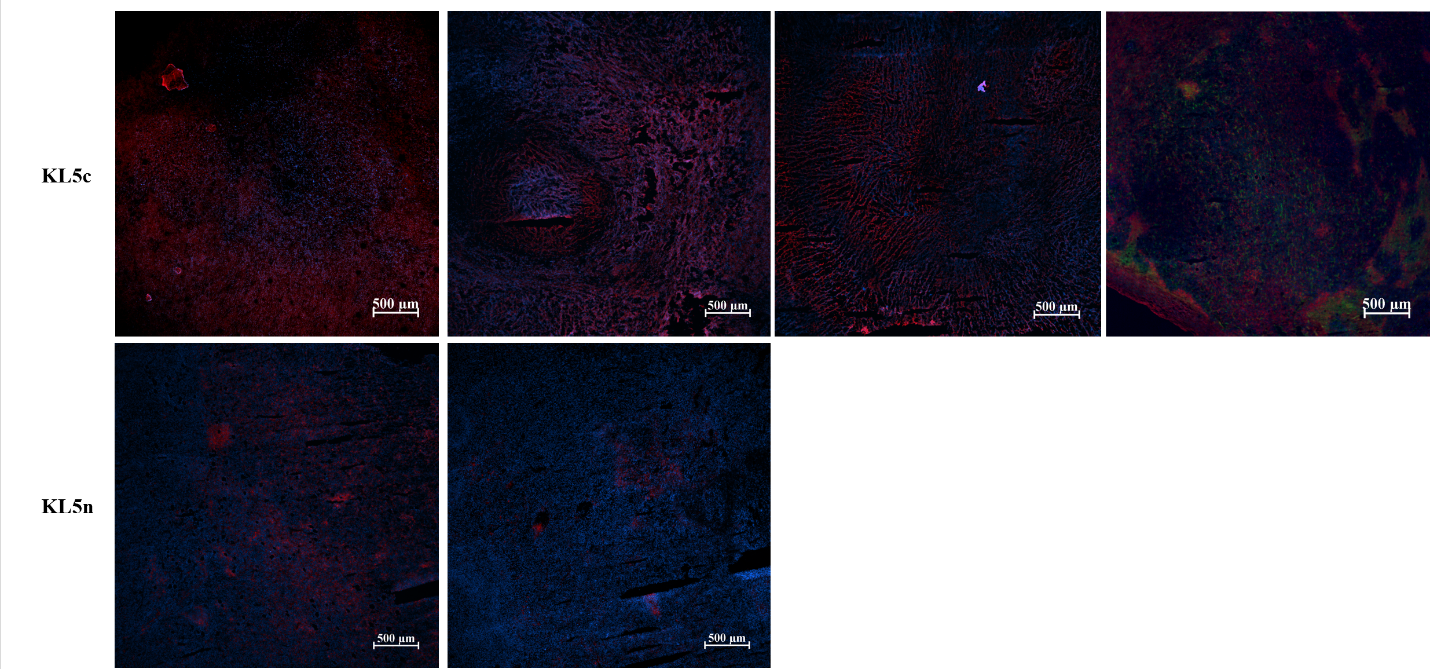

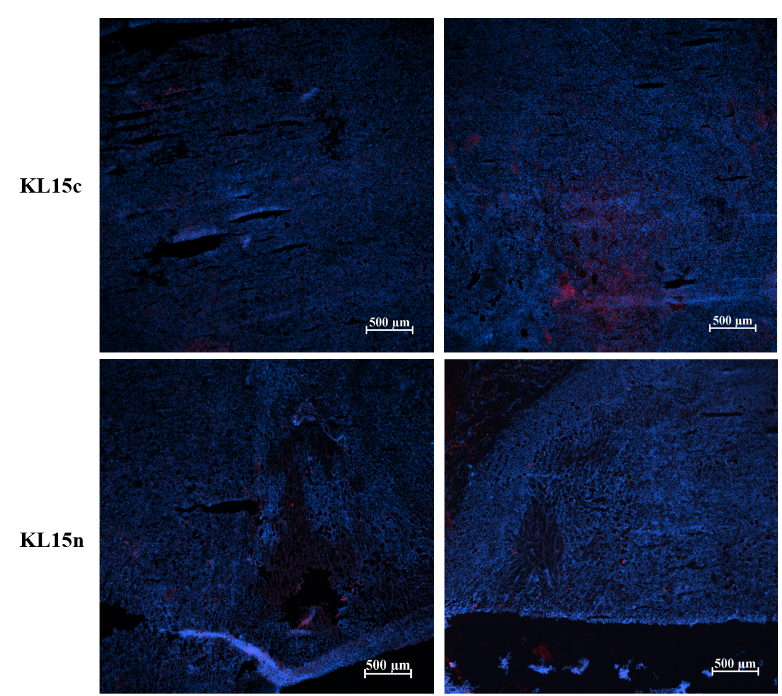


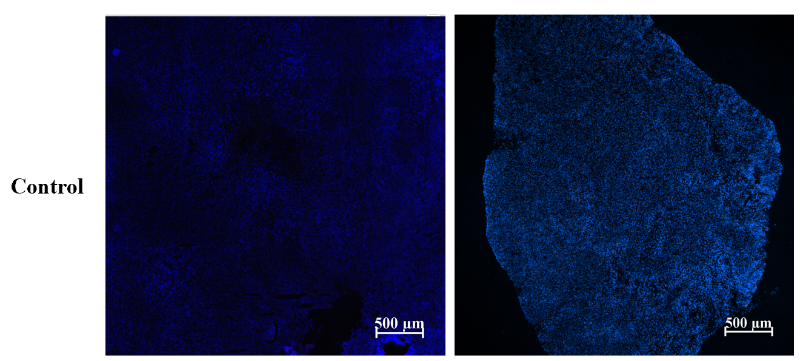


**Figure S9. KL4 shows no localization to the TME.** Each mouse was injected with a mixture of both 50 µg of functional betabody (KL5c/n or KL15c/n) and 50 µg of KL4-Biotin. KL4 was stained for in the same tissue sections as the functional betabodies. Negative control KL4 was stained for using Streptavidin-Cy3 in the same tissues section as KL5c/n or KL15c/n. Separate channels for Hoescht and Cy3 were used to visualize KL4 in the same images from Figure S8. Here the channel for KL5c/n or KL15c/n (Cy5) was excluded. The same images were used in order to show simultaneous localization of the functional betabodies and lack of localization of the negative control.


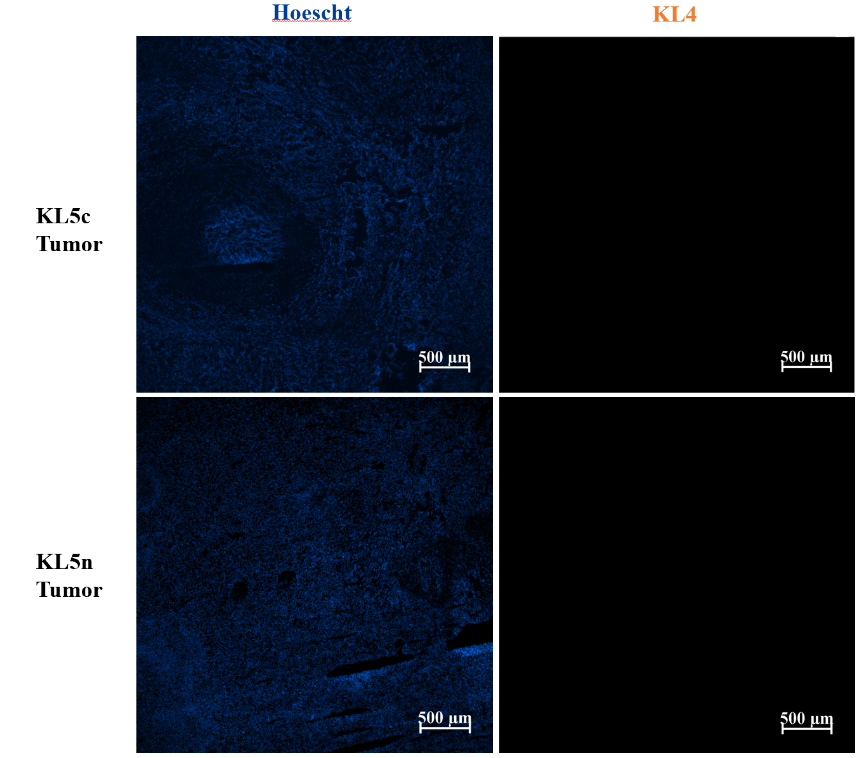


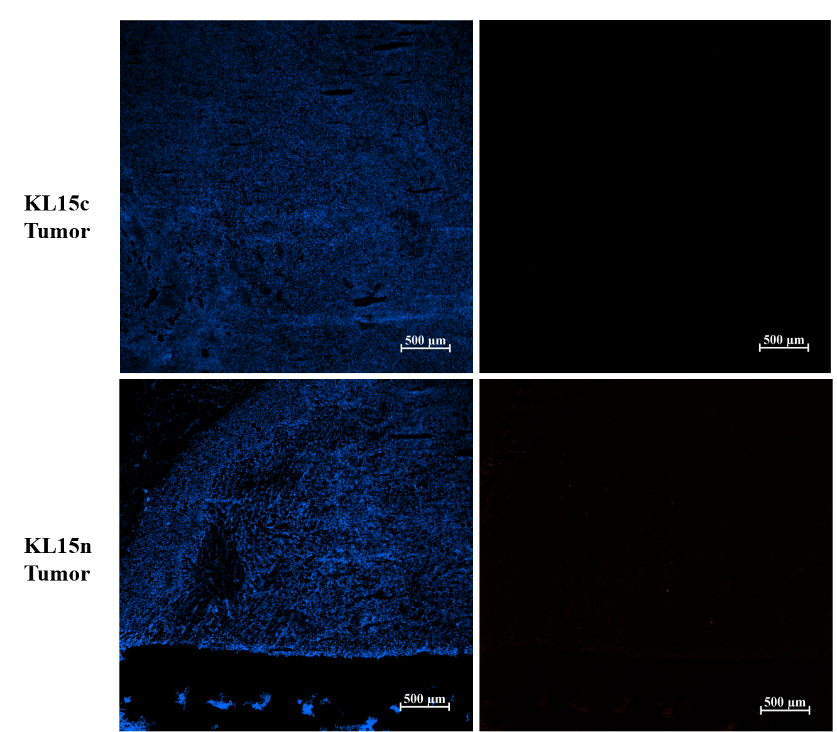


**Figure S10. Organs stained for betabody showed no localization outside of 4T1 metastases in the lungs.** 4x images of organs from all treated mice were analyzed for betabody localization using ImageJ. Sections were stained and imaged using the same protocol as for tumor sections.


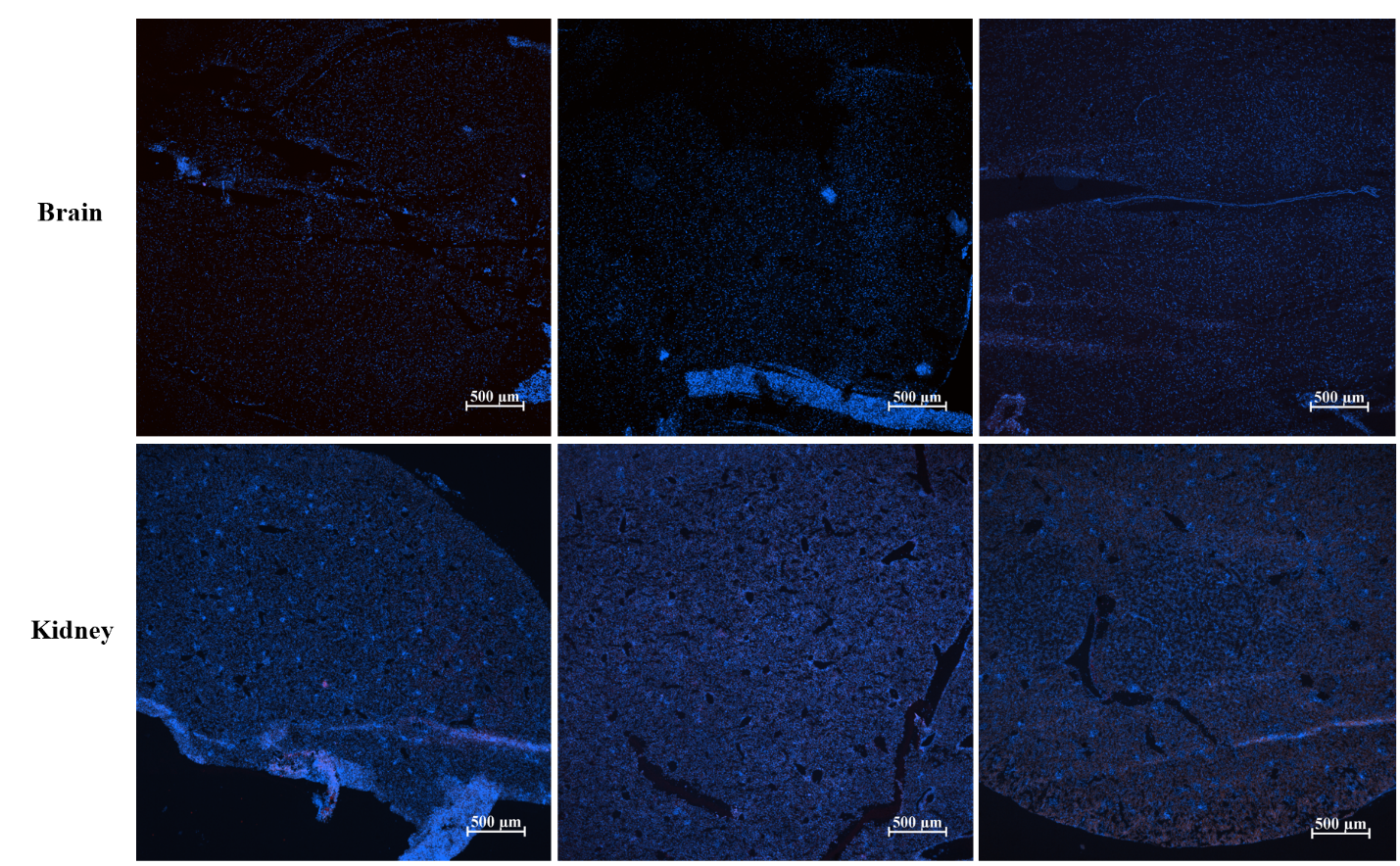

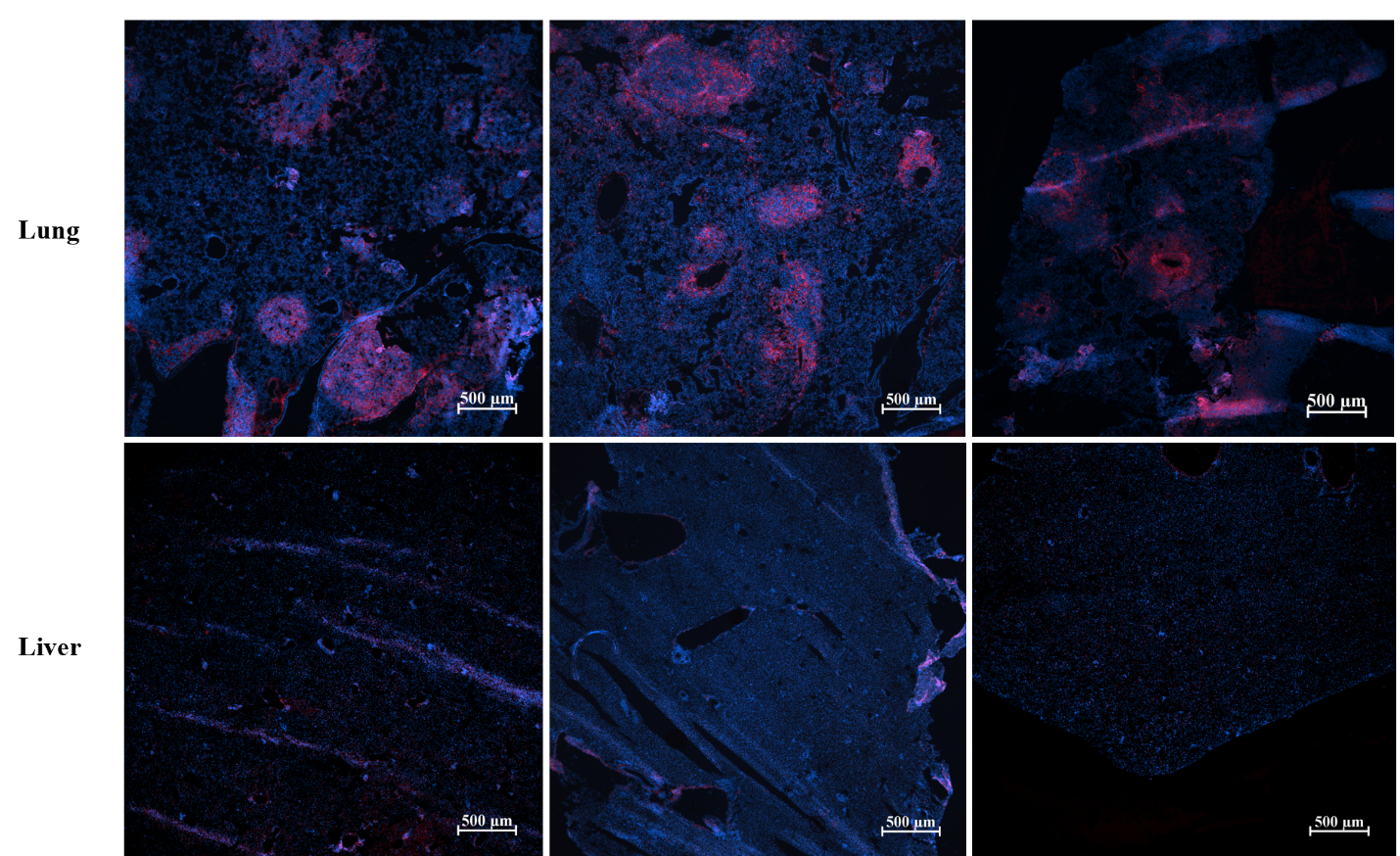


**Figure S11. Competition assay.** A. Plate was co-incubated with 25 nM Annexin V-biotin and 1 to 2 dilutions of KL5c, starting with 25 µM as the highest concentration. Annexin V binding was probed for with Streptavidin-HRP. Conditions were plated in triplicate. B. Plate was co-incubated with 25 nM KL5c and 1 to 2 dilutions of Annexin V, starting with 5 µM as the highest concentration. KL5c binding was probed for with anti-mouse Fcγ specific-HRP. Conditions were plated in triplicate.

**
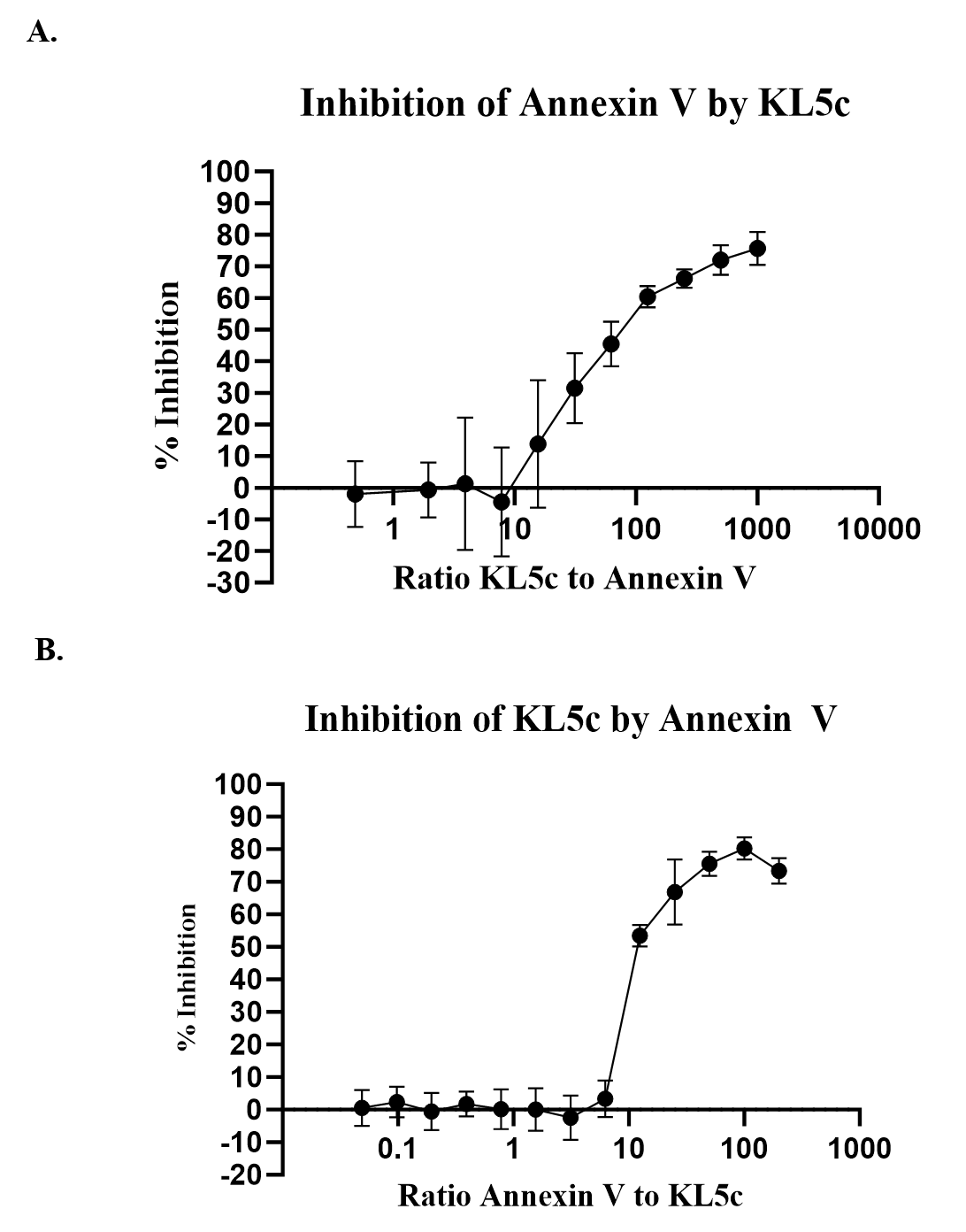
**
